# Mechanisms regulating cerebral perfusion during running exercise: Influence of foot strike‐induced pulsatility oscillations, CO_2_ reactivity and BP

**DOI:** 10.1113/JP288117

**Published:** 2026-09-01

**Authors:** Lydia L. Simpson, Dean R. Perkins, Connor A. Howe, Christina D. Bruce, William K. Cornwell, Justin S. Lawley

**Affiliations:** ^1^ Department of Sport Science Universität Innsbruck Innsbruck Austria; ^2^ Bristol Medical School, Translational Health Sciences University of Bristol Bristol UK; ^3^ Department of Medicine‐Cardiology University of Colorado Anschutz Medical Campus Aurora Colorado USA; ^4^ Clinical Translational Research Center University of Colorado Anschutz Medical Campus Aurora Colorado USA

**Keywords:** cerebrovascular reactivity, exercise, middle cerebral artery, pulsatility oscillations, running, transcranial Doppler

## Abstract

**Abstract:**

During running, cerebral blood flow (CBF) does not exhibit the inverted‐U response observed during cycling; instead, CBF increases up to maximal exercise, despite reductions in end‐tidal CO_2_ ($ET_{CO_{2}}$). The mechanism(s) driving this divergent response are unknown but may relate to foot strike‐induced pulsatility oscillations and/or reductions in cerebrovascular CO_2_ reactivity (CVR). We assessed middle cerebral artery blood velocity (MCA_v_), $ET_{CO_{2}}$ and MCA_v_ pulsatility variability during an incremental running test (*n* = 43) and fixed workload exercise under two conditions (*n* = 17): (1) free running and (2) diastolic stepping, where the foot‐strike was synced to the diastolic phase of the cardiac cycle. CVR was assessed (*n* = 12) at rest and during running. Young, healthy males and females exhibited progressive increases in the MCA_v_ mean during incremental running (Females, Rest: 49.9 ± 11.3 cm s^−1^, ${\dot{V}}_{O_{2}\max}$: 65.7 ± 11.7 cm s^−1^; Males, Rest: 45.9 ± 10.8 cm s^−1^, ${\dot{V}}_{O_{2}\max}$: 67.1 ± 13.0 cm s^−1^; ANOVA *P *< 0.0001) despite reductions in $ET_{CO_{2}}$ at higher intensities (Females, Rest: 36.41 ± 5.1 mmHg, ${\dot{V}}_{O_{2}\max}$: 28.2 ± 5.1 mmHg; Males, Rest: 38.6 ± 4.5 mmHg; ${\dot{V}}_{O_{2}\max}$: 32.5 ± 9.0 mmHg; ANOVA *P *< 0.0001). Diastolic stepping removed pulsatility oscillations, reducing MCA_v_ pulsatility variability (0.14 ± 0.04 mmHg), compared with free running (0.29 ± 0.09 mmHg, *P *< 0.0001). However, the MCA_v_ mean was not different (56.1 ± 8.1 cm s^−1^ *vs*. 58.0 ± 8.7 cm s^−1^; *P = *0.211). CVR to hypocapnia decreased from rest to running (−0.79 ± 0.44 *vs*. ‐0.19 ± 0.50 cm s^−1^ mmHg^−1^; *P = *0.006). Additional experiments found that hypocapnic CVR progressively decreased with increasing exercise intensity (ANOVA *P* = 0.0065), related to the increase in arterial pressure, but was similar during running and cycling when arterial pressure was matched (*P* = 0.999). These findings demonstrate that the CBF response during intense exercise is related to the prevailing arterial pressure and not modality *per se*, likely reflecting cerebrovascular vasoconstrictor reserve.

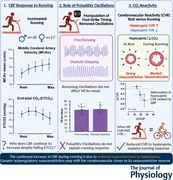

**Key points:**

- Middle cerebral artery velocity (MCA_v_) increases progressively during incremental running, contrasting with the inverted‐U response observed during cycling.
- We investigated whether this divergent response was explained by foot strike‐induced pulsatility oscillations or altered cerebrovascular CO_2_ reactivity (CVR).
- Removing pulsatility oscillations, by synchronizing foot‐strike with the cardiac cycle, had no effect on the MCA_v_ mean, indicating that pulsatility oscillations do not explain the continued increase in MCA_v_ during running.
- In contrast, hypocapnic CVR was markedly attenuated during high‐intensity running. Follow‐up experiments demonstrated that hypocapnic vasoconstrictor reserve progressively declined with increasing exercise intensity and was related to the increase in mean arterial pressure (MAP) but was similar during running and cycling when MAP was matched.
- These findings show that the progressive increase in MCA_v_ during running is facilitated by a reduced responsiveness to hypocapnia, which appears related to the prevailing arterial pressure rather than modality *per se*. This attenuation likely reflects a reduced cerebrovascular vasoconstrictor reserve.

## Graphical abstract

**Abstract figure legend** Young, healthy males and females completed an incremental running test, demonstrating a progressive increase in middle cerebral artery blood velocity (MCA_v_) up to maximal exercise despite reductions in end‐tidal CO_2_. To investigate the underlying mechanism, foot‐strike timing was synchronized to the diastolic phase of the cardiac cycle using auditory pacing, which removed pulsatility oscillations but did not affect the MCA_v_ mean. Cerebrovascular CO_2_ reactivity (CVR) was assessed at rest and during high‐intensity running, showing an enhanced reactivity to hypercapnia but a blunted reactivity to hypocapnia. Additional experiments demonstrated that hypocapnic CVR progressively declined with increasing exercise intensity, which was related to the change in mean arterial pressure (MAP), with no difference between CVR during running and cycling when matched for MAP. Together, these findings suggest that as arterial pressure rises during exercise, the cerebral circulation likely operates closer to its vasoconstrictor limit, reducing its capacity for further vasoconstriction in response to hypocapnia. This attenuated hypocapnic responsiveness likely explains the continued rise in MCA_v_ during running and may be less evident during cycling due to differences in arterial pressure rather than modality *per se*.

## Introduction

Precise control of cerebral blood flow (CBF) is essential to ensure adequate oxygen and substrate delivery and maintenance of acid‐base homeostasis. During exercise, middle cerebral artery blood velocity (MCA_v_; an index of regional CBF) increases up to ∼60% of ${\dot{V}}_{O_{2}\max}$, followed by a progressive decrease towards baseline as exercise intensity continues to increase (Jorgensen et al. 1992; Linkis et al. 1995; Moraine et al. 1993). Several factors contribute to CBF regulation during exercise, including arterial blood gases, cerebral metabolism, cardiac output, cerebral autoregulation and neural mechanisms (reviewed by Smith & Ainslie 2017). The initial increase in MCA_v_ is thought to be primarily mediated by regional increases in neuronal activity within the motor‐sensory cortex and supplementary motor area, allowing matching of blood flow to cerebral metabolic demand (i.e. neurovascular coupling (Jorgensen et al. 1992; Linkis et al. 1995)). However, the decrease in MCA_v_ at higher exercise intensities is primarily driven by hyperventilation‐induced hypocapnia and resulting cerebral vasoconstriction (Olin et al. 2011; Subudhi et al. 2011). Notably, most of this research has been performed during cycling exercise, where the measurement of CBF is arguably less challenging but overlooks the influence of exercise modality on these responses (Edwards et al. 2002; Furlong et al. 2020; Lyngeraa et al. 2013; Pott et al. 1997). Furlong et al. (2020) recently reported that ‘high fit’ individuals show a biphasic response during incremental cycling exercise but a progressive increase in MCA_v_ during incremental running (up to 95% ${\dot{V}}_{O_{2}\max}$). This occurred despite comparable reductions in end‐tidal CO_2_ ($ET_{CO_{2}}$) with both modalities, leaving the mechanisms responsible for the divergent CBF response in running unclear.

One possibility for the continued rise in CBF during running is that an additional factor opposes and masks the vasoconstrictive effects of reductions in CO_2_. Notably, a distinctive oscillatory pattern is observed in the MCA_v_ profile during running, where cyclic oscillations in pulsatility occur, which do not occur during cycling (Furlong et al. 2020; Lyngeraa et al. 2013). This oscillatory pattern originates in the arterial system due to beat‐to‐beat oscillations in arterial pulse pressure, referred to as the beat phenomenon (Leahy et al. 2024; Palatini et al. 1989; Rowell et al. 1968). These oscillations are thought to result from the relative timing and interaction of two waves; the forward travelling, cardiac‐generated pressure wave, and the opposing backward travelling inertia pressure wave generated from the foot‐strike, which influence stroke volume (Wakeham et al. 2023). One suggested hypothesis from the literature is that these oscillations may challenge cerebral autoregulation (Furlong et al., 2020; Lyngerra et al., 2013), leading to the cerebrovasculature becoming more pressure‐passive during running.

Cerebral autoregulation, which dampens the effects of exercise‐induced increases in blood pressure (BP) on CBF, is reported to be largely maintained during cycling exercise (Brys et al. 2003). However, running presents a unique challenge. Foot strike‐induced BP oscillations are reported to be between 0.3 and 0.7 Hz (Lyngeraa et al., 2013), which are above the frequency where dynamic cerebral autoregulation is considered effective (Hamner et al. 2004; Hamner et al. 2019; Tan 2012; Zhang et al. 1998). Thus these rapid BP oscillations appear to occur too fast to be effectively buffered. As a result, CBF may increasingly follow increases in BP (i.e. perfusion pressure) during running exercise, as they are no longer offset by dynamic autoregulatory vasoconstriction. These oscillations may also have downstream effects on shear‐mediated mechanisms. Nevertheless, the influence of arterial pulsatility oscillations on CBF responses to running has not been experimentally investigated.

Another possibility for the continued increase in MCA_v_ with running is a reduced cerebrovascular responsiveness to changes in CO_2_ (i.e. cerebrovascular CO_2_ reactivity), where a given reduction in $ET_{CO_{2}}$ elicits less cerebral vasoconstriction, and an attenuated decrease in MCA_v_. While CO_2_ reactivity is reported to be maintained or even augmented during low‐ to moderate‐intensity cycling exercise, compared with rest (Murrell et al. 2013; Nowak‐Flück et al. 2018; Ogoh et al. 2008; Rasmussen et al. 2006), CO_2_ reactivity during high‐intensity running exercise has never been assessed.

Thus, the aims of Study 1 were to: (1) characterize the cerebrovascular responses to incremental running exercise; (2) determine the influence of beat‐to‐beat pulsatility oscillations on cerebrovascular responses to running by manipulating foot‐strike timing; and (3) assess cerebrovascular CO_2_ reactivity at rest and during running. To address aim 1, we assessed changes in MCA_v_ during an incremental running test to exhaustion. To address aim 2, we assessed MCA_v_ and beat‐to‐beat BP during treadmill running with and without manipulation of foot‐strike timing, to remove pulsatility oscillations. To address aim 3, we assessed MCA_v_ reactivity to both hypercapnia and hypocapnia at rest and during running exercise. We hypothesized that: (1) the MCA_v_ mean would increase progressively up to maximal running exercise, replicating the findings of Furlong et al. (2020); (2) syncing the foot‐strike to either the diastolic or systolic phase of the cardiac cycle would remove pulsatility oscillations and decrease the MCA_v_ mean *versus* free running; and (3) cerebrovascular CO_2_ reactivity would be comparable to rest, or increased, during running, consistent with previous reports in cycling (Ogoh et al. 2008; Rasmussen et al. 2006).

Results from Study 1 showed that MCA_V_ reactivity to hypocapnia was not only blunted during high‐intensity running, but practically absent. Thus, we developed an alternative working hypothesis. We hypothesized that reduced responsiveness to hypocapnia reflects the cerebrovasculature operating close to its vasoconstrictor reserve, determined by prevailing arterial pressure and thus the magnitude of autoregulatory vasoconstriction. To examine this hypothesis, we had two further aims: (4) compare cerebrovascular CO_2_ responsiveness during cycling and running when matched for BP, and ‘autoregulatory load’; and (5) determine the relationship between exercise intensity and BP on cerebrovascular CO_2_ responsiveness to hypocapnia. To address aim 4, we assessed MCA_v_ reactivity to hypocapnia during cycling and running during steady‐state workloads when modalities were matched for MAP (i.e. perfusion pressure). To address aim 5, we assessed MCA_v_ reactivity to hypocapnia at rest and during cycling exercise at three steady‐state fixed workloads. We hypothesized that cerebrovascular CO_2_ reactivity would be independent of modality and comparable during running and cycling when matched for the prevailing BP, and that cerebrovascular CO_2_ responsiveness would progressively decrease with increasing exercise intensity, and this would be associated with the magnitude of increase in BP as a greater proportion of the available cerebrovascular vasoconstrictor reserve is recruited by static autoregulation.

## Methods

### Ethical approval

All experimental procedures were approved by the Ethical Review Committee at the University of Innsbruck (ERN_17‐1570) and conformed to the latest revision of the *Declaration of Helsinki*, except for registration in a database. All participants provided verbal and written informed consent before participation.

### Experimental design and participants

This investigation consisted of two independent series of studies performed in separate cohorts, with experiments completed in the Physiology Laboratory at Innsbruck University. Study 1 consisted of three protocols to address aims 1, 2 and 3: Protocol 1 – assessment of MCA_v_ responses to incremental treadmill running in males and females; Protocol 2 – manipulation of foot‐strike timing; and Protocol 3 – assessment of cerebrovascular CO_2_ reactivity. Forty‐three healthy, physically active males and females completed Protocol 1, with a subset of these participants also completing Protocol 2 (*n* = 17) and Protocol 3 (*n* = 12), for which the sample sizes were based on *a priori* power calculations. Following completion of Study 1, Study 2 was conducted to further investigate the role of BP in CO_2_ responsiveness during exercise. Study 2 consisted of a further two protocols to address aims 4 and 5: Protocol 4 – comparison of MCA_v_ reactivity during cycling and running exercise at a matched BP (*n* = 6); and Protocol 5 – effect of exercise intensity and blood pressure on MCA_v_ reactivity (*n* = 5).

Participant demographics for all protocols are shown in Table 1. All participants were non‐smokers, free from cardiovascular, respiratory, metabolic and cerebrovascular disease, and were not taking any prescription or over‐the‐counter medication at the time of participation, apart from oral hormonal contraceptives. All female participants were tested in the first five days of their menstrual cycle or during the placebo phase of hormonal contraception use. All participants were instructed to abstain from caffeine for 12 h and alcohol and vigorous exercise for at least 24 h before the experimental session and arrive at the laboratory having consumed a light meal at least 2 h before.

**Table 1:** Participant demographics

		Study 1		Study 2	
	Protocol 1 (*n* = 43)	Protocol 2 (*n* = 17)	Protocol 3 (*n* = 12)	Protocol 4 (*n* = 6)	Protocol 5 (*n* = 5)
Males/females	26/17	13/4	8/4	3/3	2/3
Age (years)	27 ± 4	26 ± 5	27 ± 5	28 ± 4	28 ± 4
Height (cm)	175.6 ± 8.4	178.4 ± 8.3	178.2 ± 9.5	173.5 ± 3.9	172.6±3.7
Weight (kg)	69.9 ± 9.7	73.4 ± 10.6	74.6 ± 10.3	66.5 ± 5.4	64.8 ± 4.2
${\dot{V}}_{O_{2}\max}$ (ml kg^−1^ min^−1^)	58.5 ± 10.2	62.2 ± 7.7	60.7 ± 9.6	–	–

#### Study 1

##### Protocol 1 – Assessment of MCA_v_ responses to incremental running

Following arrival at the laboratory, participants were instrumented with equipment to measure cerebrovascular, cardiovascular, ventilatory and metabolic parameters (described in Measurements). Following a period of stabilization, 5 min of resting data in the standing position were obtained to determine baseline values. Participants then performed an incremental exercise test on a treadmill (HP Cosmos, Pulsar, Germany) until volitional exhaustion. The incremental test commenced at a speed of 8 km h^−1^ with a 0% incline, and speed was increased by 1 km h^−1^ every minute until 12 km h^−1^, thereafter the incline was increased by 1% every 30 s. Following a period of recovery, a verification test was performed to confirm the attainment of the maximal rate of oxygen consumption (${\dot{V}}_{O_{2}\max}$).

#### Assessment of mechanisms regulating MCA_v_ responses to running

##### Protocol 2 – Manipulation of foot‐strike timing

Participants were familiarized with the protocol and practised matching their foot‐strike to an auditory tone while running. Participants then ran on a treadmill at a fixed, individualized pace under three conditions: (1) free running, (2) diastolic stepping, and (3) systolic stepping, each for 3 min, in a randomized order. Each condition was separated by a period of recovery. The individualized pace was chosen to: (1) corresponded to the workload where heart rate and stride frequency are similar; and (2) corresponded to an intensity where $ET_{CO_{2}}$ is reduced and MCA_v_ would be expected to decrease (i.e. where MCA_v_ responses to running and cycling exercise diverge). The average intensity was 70.9 ± 9.0% ${\dot{V}}_{O_{2}\max}$. Systolic and diastolic stepping conditions involved participants matching their foot‐strike to an auditory tone occurring at either 45% R–R interval (diastolic stepping condition) or the subject's R‐wave (systolic stepping condition), to synchronize their foot‐strike to the diastolic or systolic phase of the cardiac cycle, respectively. During free running, participants ran without any auditory guidance. A subset of participants (*n* = 11) also performed a fourth condition (cognitive effort matched condition), where participants matched their foot‐strike to a randomly generated auditory tone. The auditory tone occurred at random across the cardiac cycle (i.e. sometimes during diastole, sometimes during systole), comparable to free running. This condition was designed to match the neural activation associated with the cognitive demand of matching foot‐strike to a metronome, which may independently alter CBF.

##### Protocol 3 – Assessment of cerebrovascular CO_2_ reactivity

Cerebrovascular reactivity to both hypocapnia and hypercapnia was assessed at rest and during exercise, as hysteresis is observed in cerebrovascular CO_2_ reactivity (Ogoh et al. 2008; Rasmussen et al. 2006). Following the achievement of rested, standing steady‐state $ET_{CO_{2}}$, participants hyperventilated to reach a reduced $ET_{CO_{2}}$ comparable to that observed at maximal exercise, determined during the incremental exercise test (Protocol 1). Once the desired $ET_{CO_{2}}$ was achieved, participants maintained the $ET_{CO_{2}}$ constant for a further 30 s before returning to normal tidal breathing. Following a period of recovery, CO_2_ was added to the inspired air by manually titrating flow until $ET_{CO_{2}}$ was increased to match the magnitude of decrease observed with prior hyperventilation. Once the desired $ET_{CO_{2}}$ was achieved, $ET_{CO_{2}}$ was held at a steady state for 30 s before participants were returned to breathing room air. Following a further period of recovery, participants exercised at a fixed, individualized workload before repeating the cerebrovascular reactivity protocol. The individualized workload corresponded to 76 ± 4% ${\dot{V}}_{O_{2}\max}$ and importantly is an intensity whereby $ET_{CO_{2}}$ is reduced during fixed‐intensity steady‐state exercise and MCA_v_ would be expected to decrease (i.e. where MCA_v_ responses to running and cycling exercise diverge).

#### Study 2

##### Protocol 4 – Comparison of cerebrovascular responsiveness to hypocapnia between running and cycling at matched BP

To determine whether cerebrovascular responsiveness differed between exercise modalities when matched for BP, participants completed separate running and cycling trials consisting of progressive 3 min stages of steady‐state exercise. During the first visit, participants initially performed paced hyperventilation at rest to reduce $ET_{CO_{2}}$ to ∼20 mmHg, a level associated with near‐maximal cerebral vasoconstriction (Harper & Glass, 1965). Participants then completed progressive 3 min stages of steady‐state running exercise, with speed increased by 2 km h^−1^ every stage. At the end of each steady‐state stage, participants repeated the hyperventilation procedure. On a separate visit, participants completed a near‐identical protocol during cycling exercise. However, cycling power output was adjusted at each stage to match the rise in mean arterial pressure (MAP) during each stage to those observed during the running trial, to match for autoregulatory load. Once a steady state had been achieved, participants repeated the same hyperventilation procedure. For analysis, data were extracted from the highest exercise stage completed by each individual at which BP was closely matched between modalities and transcranial Doppler signals were well maintained throughout both exercise and hyperventilation.

##### Protocol 5 – Effect of exercise intensity and BP on cerebrovascular responsiveness to hypocapnia

To determine whether cerebrovascular CO_2_ responsiveness to hypocapnia differs with exercise intensity and prevailing BP, data were extracted from three stages of the incremental cycling protocol from Protocol 4, to obtain a graded elevation in mean arterial BP. Cycling was selected because the measurement of beat‐by‐beat BP and transcranial Doppler is less challenging. The absolute workload differed between participants, with the three stages corresponding to mean workloads of 64 ± 18, 105 ± 8.4 and 155 ± 36 W. At each stage, once steady state had been reached, participants repeated the hyperventilation procedure.

### Measurements

#### Cardiovascular variables

Heart rate was continuously recorded via Bluetooth polar chest belt (Wear link, Polar Electro, Finland) during Protocols 1, 4 and 5, and electrocardiogram during Protocols 2 and 3 (ECG: Lead II). During manipulation of foot‐strike timing, beat‐by‐beat BP was recorded using finger photoplethysmography (Portapres, Finapres Medical Systems B.V, Amsterdam, Netherlands), with the arm placed in a sling and suspended at the level of the heart, to minimize movement artefacts. Portapres values were calibrated at rest against brachial artery BP measurements taken from the right arm by electrosphygmomanometry (Tango, M2, SunTechMedical Instruments Inc., USA).

#### Cerebrovascular variables

Middle cerebral artery blood velocity (MCA_v_) was measured via transcranial Doppler (TCD) ultrasound (Spencer Technologies, Seattle, WA, USA) and used as an index of cerebral perfusion. A 2 MHz TCD probe insonated the right middle cerebral artery (MCA) via the temporal window above the zygomatic arch, with search and identification procedures performed in accordance with established guidelines (Willie et al. 2011). Once an acceptable signal was found, the TCD probe was fixed with a specialized headband (model M600 bilateral head frame, Spencer Technologies) and the face mask strapping fitted over the top of the headset to help maintain a fixed position throughout exercise.

#### Ventilatory and metabolic parameters

Respiratory flow, tidal volume, respiratory rate, and minute ventilation were measured via a heated pneumotachograph on the expiratory line (Hans Rudolph Inc, USA). Subjects wore a facemask and breathed through a two‐way valve (Hans Rudolph Inc, USA) connected to a 4.7 L gas mixing chamber (AD Instruments, UK, MLA246) to collect expired gases. Fractional concentrations of O_2_ and CO_2_ in the expired air were determined using a respiratory gas analyser (AD Instruments, UK, ML206), calibrated before each measurement and used to calculate ${\dot{V}}_{O_{2}}$ and ${\dot{V}}_{CO_{2}}$. Breath‐by‐breath end‐tidal CO_2_ ($ET_{CO_{2}}$) and O_2_ (ETO_2_) were sampled at the mouth and recorded by a respiratory gas analyser (AD Instruments, UK, ML206).

#### Foot‐strike synchronization

Participants wore a chest strap with an ECG sensor and accelerometer attached that detected the R‐wave and foot‐strike signals. The foot‐strike was defined as the point when the 3‐axis accelerometer signal crossed 1 *g* in a positive direction during each gait cycle. These signals were streamed wirelessly to a step‐timing guidance software (Counterpace software application running in iOS, Pulson, Inc., Palo Alto, CA), which was used to generate the auditory tone at the given percentage of the individual's R–R interval and determine the timing of each foot‐strike, relative to the R‐R interval. The auditory tone was played via wireless headphones so it could be easily heard by the participant running on the treadmill. Correct step timing was determined via the step‐guidance software (Counterpace).

## Data analysis

Cardiovascular, cerebrovascular, ventilatory and metabolic variables were sampled continuously at 1 kHz via an analogue‐to‐digital converter (Powerlab, 16/30, ADInstruments, Colorado Springs, CO, USA) and stored for offline analysis. For Protocol 1, 30 s averages of cardiovascular, cerebrovascular, ventilatory and metabolic parameters were taken at rest, 40%, 60%, 80% and 100% of ${\dot{V}}_{O_{2}\max}$ during the incremental exercise test. ${\dot{V}}_{O_{2}\max}$ was taken as the highest 30 s average for ${\dot{V}}_{O_{2}}$. For Protocol 2, beat‐by‐beat MCA pulsatility was calculated as ((MCA_v_ systolic ‐ MCA_v_ diastolic)/MCA_v_ mean), and MCA_v_ pulsatility variability was calculated as the standard deviation (SD) over the final 30 s of each stepping condition. MCA pulsatility variability was used to numerically quantify the beat‐by‐beat oscillations in pulsatility during running (Fig. 1) and confirm whether foot‐strike manipulation successfully reduced pulsatility oscillations. Blood pressure and pulse pressure variability were also calculated using the same approach. An index of cerebrovascular conductance (CVC) was calculated as MCA_v_ mean/mean BP. For Protocols 3, 4 and 5 cerebrovascular reactivity was calculated as the ratio of ∆MCA_v_ mean/∆$ET_{CO_{2}}$ using the values immediately preceding the test and the peak response.

**Figure 1:**
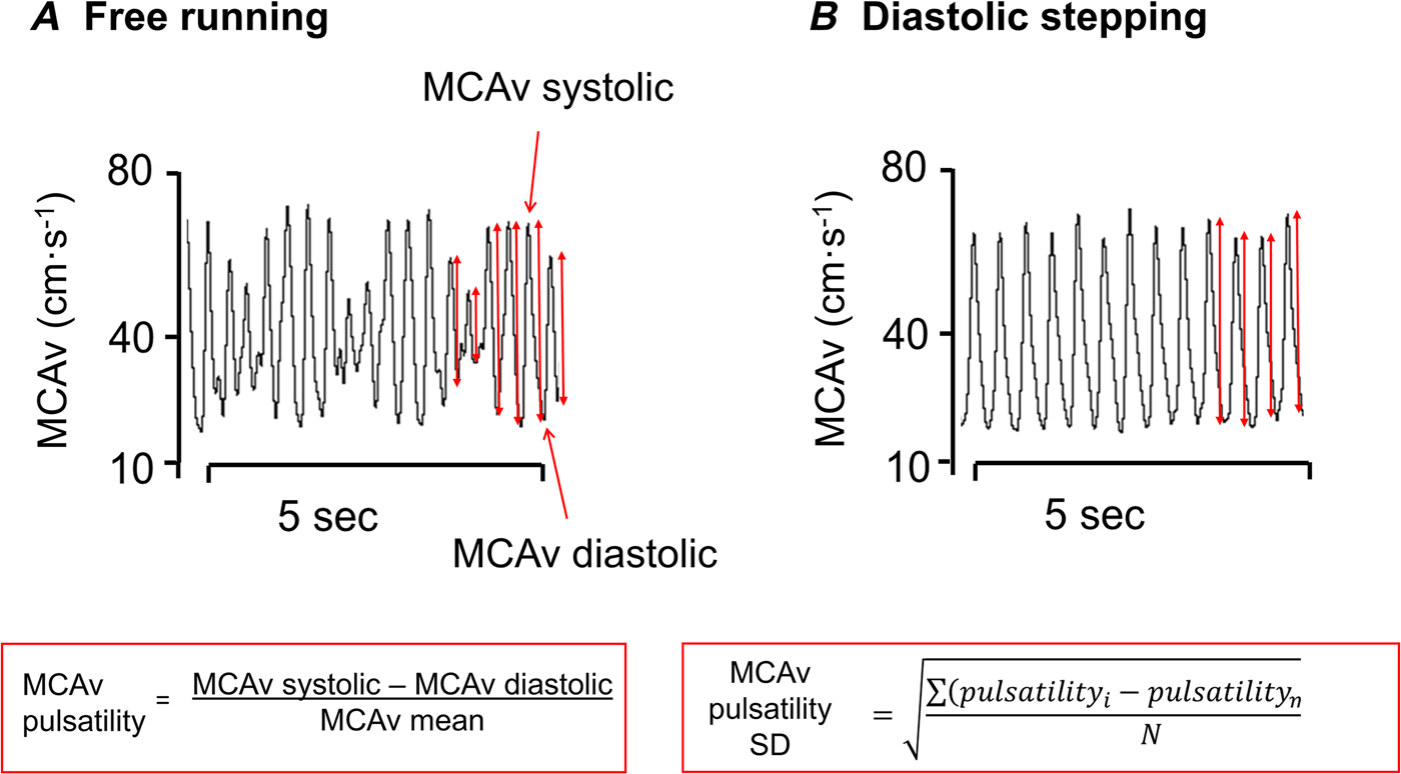
Calculation of beat‐by‐beat middle cerebral artery (MCA) pulsatility variability MCA_v_ pulsatility was calculated for every cardiac cycle over the final 30 s of each stepping condition. The standard deviation of the beat‐by‐beat MCA pulsatility for each participant was used as an index of the variability. *pulsatility*_i_: each individual beat‐by‐beat measurement, *pulsatility_n_*: mean pulsatility, and *N* corresponds to the number of cardiac cycles.

## Statistical analyses

To address aim 1, cerebrovascular, cardiovascular and ventilatory responses to incremental running exercise were assessed using a two‐way ANOVA, with exercise intensity (Rest, 40% ${\dot{V}}_{O_{2}\max}$, 60% ${\dot{V}}_{O_{2}\max}$, 80% ${\dot{V}}_{O_{2}\max}$, 100% ${\dot{V}}_{O_{2}\max}$) and sex (male, female) as main factors (*n* = 43). Bonferroni *post hoc* comparisons were performed. While investigation of sex differences was not a primary aim of the study, we separated male and females to establish whether divergent MCA_v_ responses were consistent across sexes and provide insight into potential sex‐specific responses. To address aim 2, differences in cerebrovascular and cardiovascular responses between free running and diastolic stepping were compared using paired *t* tests (*n* = 17). No statistical comparisons were performed on the systolic stepping condition due to the small number of participants able to successfully match their foot‐strike to the systolic phase of the cardiac cycle. Instead, in a subset of five participants who successfully performed systolic stepping, Cohen's *d* effect sizes are reported between free running, diastolic stepping and systolic stepping conditions. Differences in cerebrovascular and cardiovascular responses were also assessed between free running and cognitive load matched running, using paired *t* tests (*n* = 11). To address aim 3, differences in MCA_v_ reactivity from rest to exercise during hypercapnia (*n* = 10) and hypocapnia (*n* = 12) were assessed independently using paired *t* tests. To address aim 4, MCA_v_ reactivity between cycling and running at matched BP were compared using one‐way repeated measures ANOVA, with Bonferroni *post hoc* comparisons (*n* = 6). To address aim 5, the effect of increasing exercise intensity and BP on MCA_v_ reactivity to hypocapnia was assessed using a one‐way repeated measures ANOVA, with Bonferroni *post hoc* comparisons (*n* = 5). In addition, to examine whether the magnitude of the BP change was associated with change in MCA_v_ reactivity across exercise intensities, a linear regression analysis was performed on mean group data between the change in MAP (∆MAP) and the corresponding change in MCA_v_ reactivity (∆MCA_v_ reactivity). All statistical analyses were performed using Prism, version 7.03 (GraphPad Software Inc., San Diego, CA, USA). *P* < 0.05 was considered statistically significant. Group data are reported as the means ± SD.

## Results

### Study 1

#### Protocol 1 – Assessment of MCA_v_ responses to incremental treadmill running

An example MCA_v_ profile during incremental exercise is shown in Fig. 2. Beat‐by‐beat oscillations in pulsatility were observed in the MCA_v_ profile during running in all participants. However, the frequency of these oscillations appeared to vary across the test and between individuals (Fig. 2). Group cardiovascular and cerebrovascular haemodynamics are shown in Table 2. MCA_v_ mean increased progressively across exercise intensities in both males and females (ANOVA *P < *0.0001), with an increase in MCA_v_ mean observed at each exercise intensity and peaking at maximal exercise (Fig. 3, Table 2). In contrast, an inverted‐U response was observed for $ET_{CO_{2}}$. In males, $ET_{CO_{2}}$ increased above rest at 40% (*P = *0.0003) and 60% ${\dot{V}}_{O_{2}\max}$ (*P = *0.0039) before returning toward resting values at 80% ${\dot{V}}_{O_{2}\max}$ (*P *> 0.999) and declining below resting values at maximal exercise (*P = *0.0047, Fig. 3, Table 2). In contrast, females exhibited little change in $ET_{CO_{2}}$ at lower exercise intensities, compared with rest (40% ${\dot{V}}_{O_{2}\max}$: *P = *0.9958, 60% ${\dot{V}}_{O_{2}\max}$. *P *> 0.999), but a reduction in $ET_{CO_{2}}$ was observed after 60% ${\dot{V}}_{O_{2}\max}$ (80% ${\dot{V}}_{O_{2}\max}$: *P = *0.045, ${\dot{V}}_{O_{2}\max}$: *P = *0.0003 *versus* rest). Nevertheless, both males and females demonstrated an uncoupling between MCA_v_ and $ET_{CO_{2}}$ (Fig. 3, Table 2).

**Figure 2:**
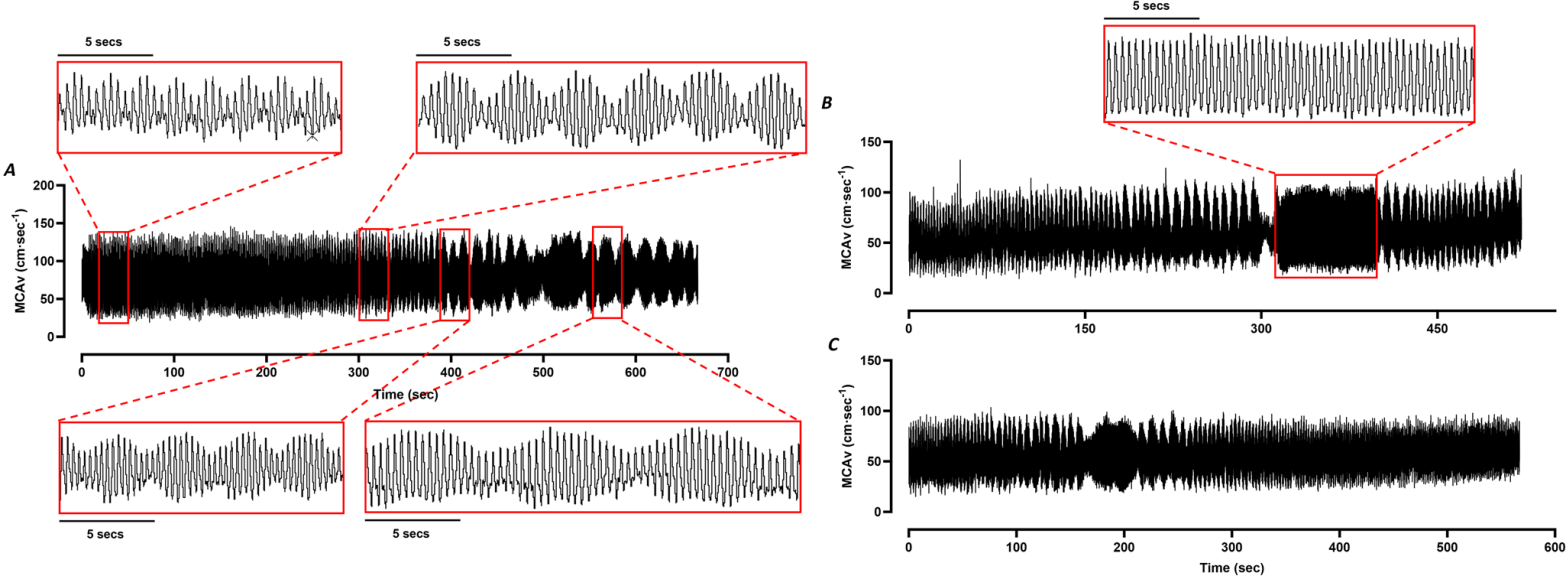
Middle cerebral artery velocity (MCA_v_) profile during incremental running exercise Data illustrate the distinctive oscillatory pattern in the MCA_v_ profile during incremental running in three participants (*A*, *B* and *C*). Pulsatility oscillations occurred in all subjects, but their frequency varied between individuals and across exercise intensities. The frequency of these oscillations has previously been related to the difference between stride frequency and heart rate (Lyngerra et al., 2013; Palatini et al., 1989). The frequency of oscillations is higher when there is a large difference between stride frequency and heart rate, with a decrease in oscillation frequency as they become more similar. For example, in participant *A* the high‐frequency oscillations during the early phases of the test decrease as the test progresses. This is likely due to the progressive increase in heart rate, which becomes increasingly similar to the participant's stride frequency. In participant *B*, the oscillation frequency also decreases as the test progresses, before these oscillations disappear. The absence of oscillations likely occurs when heart rate and stride frequency are matched, and the participant naturally syncs their foot‐strike to the diastolic phase of the cardiac cycle. However, oscillations then reappear as heart rate and stride frequency diverge once more and heart rate starts to exceed stride frequency. Therefore, different profiles observed across individuals likely result from individual differences in heart rate and stride frequency.

**Table 2:** Cerebrovascular, cardiovascular and ventilatory variables during incremental running

		Rest	40% ${\dot{V}}_{O_{2}\max}$	60% ${\dot{V}}_{O_{2}\max}$	80% ${\dot{V}}_{O_{2}\max}$	${\dot{V}}_{O_{2}\max}$	Intensity	Sex	Interaction
Heart rate (bpm)	*Males*	79 ± 10	127 ± 18*	157 ± 15*, ^†^	179 ± 11*, ^†^	188 ± 10*, ^†^	< 0.0001	0.47	0.19
	*Females*	74 ± 16	131 ± 15*	155 ± 11*, ^†^	174 ± 8*, ^†^	183 ± 8*, ^†^			
MCA_v_ mean (cm s^−1^)	*Males*	45.9 ± 10.8	55.3 ± 11.3*	61.1 ± 11.8*, ^†^	64.1 ± 12.4*, ^†^	67.1 ± 13.0*, ^†^	< 0.0001	0.88	0.0002
	*Females*	49.9 ± 11.3	55.8 ± 11.0*	58.6 ± 10.7*, ^†^	60.8 ± 11.1* ^†^	65.7 ± 11.7*, ^†^			
MCA_v_ systolic (cm s^−1^)	*Males*	71.7 ± 16.3	103.4 ± 18.9*	112.1 ± 20.6*, ^†^	113.7 ± 20.1*	113.2 ± 20.0*	< 0.0001	0.44	< 0.0001
	*Females*	73.2 ± 16.5	100.8 ± 21.1*	101.0 ± 22.5*	104.1 ± 19.1*, ^†^	105.0 ± 20.3*			
MCA_v_ diastolic (cm s^−1^)	*Males*	34.2 ± 8.4	28.3 ± 6.3*	31.0 ± 7.3*, ^†^	33.9 ± 7.9^†^	38.0 ± 9.5*, ^†^	< 0.0001	0.79	0.13
	*Females*	37.5 ± 8.1	28.9 ± 6.4*	31.3 ± 5.4*, ^†^	33.2 ± 6.2*, ^†^	37.3 ± 7.9_†_			
Ventilation (L min^−1^)	*Males*	16.2 ± 5.7	52.3 ± 8.6*	79.6 ± 11.8*	119.3 ± 20.3*	157.4 ± 26.6*	< 0.0001	< 0.0001	< 0.0001
	*Females*	9.2 ± 3.5^Ұ^	42.3 ± 11.7* ^Ұ^	59.3 ± 10.0*, Ұ	84.8 ± 12.9*, Ұ	110.0 ± 20.9*, Ұ			
$ET_{CO_{2}}$ (mmHg)	*Males*	38.6 ± 4.5	42.8 ± 5.6*	42.6 ± 6.4*	38.1 ± 7.5^†^	32.5 ± 9.0*, ^†^	< 0.0001	0.01	0.31
	*Females*	36.4±5.1	37.5 ± 4.3	37.2 ± 4.3	34.1 ± 6.1^†^	28.2 ± 5.1*, ^†^			

*Note*: Statistical comparisons were performed using two‐way repeated measures ANOVA with Bonferroni *post hoc* comparisons. Data are presented as means ± *SD*. Males *n* = 26, Females *n* = 17.

* *P* < 0.05 *versus* rest.

^†^ *P* < 0.05 *versus* previous exercise intensity.

^Ұ^ *P* < 0.05 *versus* males.

Abbreviation: MCAv, middle cerebral artery velocity.

**Figure 3:**
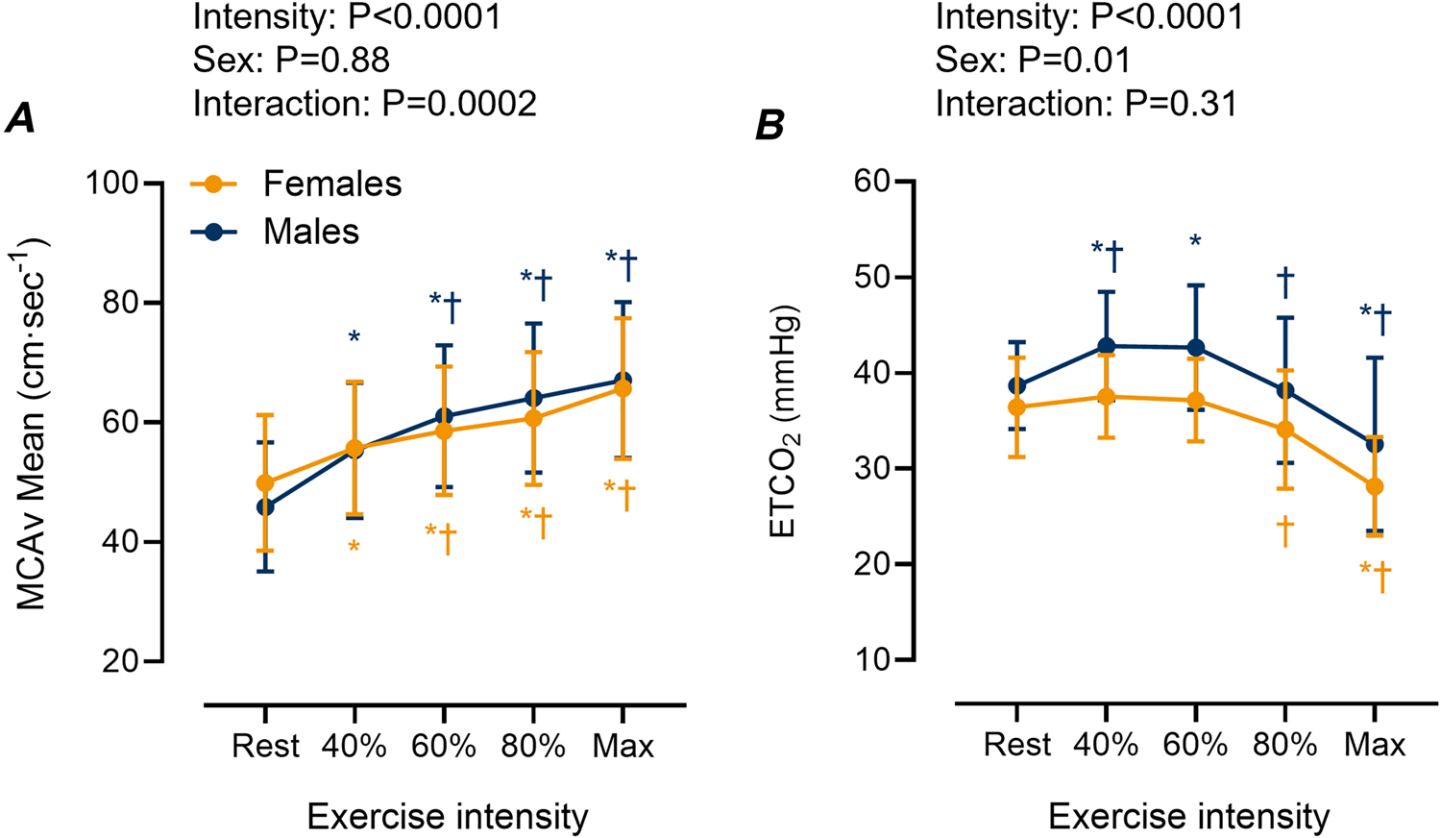
Middle cerebral artery velocity (MCA_v_) and $ET_{CO_{2}}$ responses during running incremental exercise *A*, a progressive increase in mean MCA_v_ was observed across exercise intensities. *B*, a biphasic response in $ET_{CO_{2}}$ was observed across exercise intensities. Statistical comparisons performed using two‐way ANOVA, with Bonferroni *post hoc* comparisons. Data are presented as means ± SD. *P < 0.05 *vs*. rest; ^†^ *P* < 0.05 *vs*. previous exercise intensity. Males *n* = 26, Females *n* = 17.

#### Protocol 2 – Manipulation of foot‐strike timing

Cerebrovascular and cardiovascular haemodynamics during manipulation of foot‐strike timing are presented in Table 3 and Fig. 4. Figure 4 shows an example of a representative BP and MCA_v_ profile during free running and diastolic stepping. During free running, the pulsatility oscillation frequency was 0.22 ± 0.04 Hz, with all participants exhibiting oscillation frequencies above 0.15 Hz, which is the upper range where dynamic cerebral autoregulation is effective. Syncing the foot‐strike to the diastolic phase of the cardiac cycle reduced pulse pressure variability (*P *< 0.0001) and MCA_v_ pulsatility variability (*P *< 0.0001) (Fig. 5 and Table 3), demonstrating a reduction in pulsatility oscillations *versus* free running. Importantly, despite a reduction in pulsatility oscillations, there was no difference in MCA_v_ mean between diastolic stepping and free running (*P* = 0.211, Fig. 5, Table 3). MCA_v_ systolic increased (*P = *0.001) and MCA_v_ diastolic decreased (*P = *0.0007) with diastolic stepping, resulting in an increase in average within‐beat MCA pulsatility (*P* < 0.0001) *versus* free running.

**Table 3:** Cardiovascular and cerebrovascular haemodynamics during free running and diastolic stepping conditions

	Free running (*n* = 17)	Diastolic stepping (*n* = 17)	*P* value
*Cardiovascular haemodynamics*			
Heart rate (bpm)	163 ± 6	162 ± 6	0.183
MAP (mmHg)	98.9 ± 18.6	100.7 ± 17.9	**0.008**
Systolic blood pressure (mmHg)	143.2 ± 36.7	154.8 ± 35.9	**<** **0.0001**
Diastolic blood pressure (mmHg)	75.8 ± 13.6	73.6 ± 14.0	**0.009**
Pulse pressure (mmHg)	68.6 ± 31.7	80.5 ± 32.3	**<** **0.0001**
MAP variability (mmHg)	4.2 ± 2.0	3.9 ± 2.0	0.346
Systolic blood pressure variability (mmHg)	14.7 ± 6.7	7.5 ± 4.0	**<** **0.0001**
Diastolic blood pressure variability (mmHg)	5.1 ± 2.3	4.2 ± 2.2	**0.033**
Pulse pressure variability (mmHg)	17.2 ± 7.7	7.2 ± 3.96	**<** **0.0001**
*Cerebrovascular haemodynamics*			
MCA_v_ mean (cm s^−1^)	56.1 ± 8.1	58.0 ± 8.7	0.211
MCA_v_ systolic (cm s^−1^)	99.8 ± 17.8	110.1 ± 20.1	**0.001**
MCA_v_ diastolic (cm s^−1^)	29.7 ± 4.4	25.2 ± 3.8	**0.0007**
MCA pulsatility (cm s^−1^)	1.25 ± 0.20	1.46 ± 0.19	**<** **0.0001**
MCA pulsatility variability (cm s^−1^)	0.29 ± 0.09	0.14 ± 0.04	**<** **0.0001**
CVC index (cm s^−1^ mmHg^−1^)	0.59 ± 0.16	0.57 ± 0.12	0.441
Ventilation (L min^−1^)	86.7 ± 11.5	90.0 ± 11.8	0.171
$ET_{CO_{2}}$ (mmHg)	32.8 ± 5.0	32.1 ± 4.7	0.308

*Note*: Statistical comparisons performed using paired *t* tests. Data are presented as means ± SD.

Abbreviations: CVC, cerebrovascular conductance; $ET_{CO_{2}}$, end‐tidal CO_2_; MAP, mean arterial pressure; MCA, middle cerebral artery; MCA_v_, middle cerebral artery velocity.

**Figure 4:**
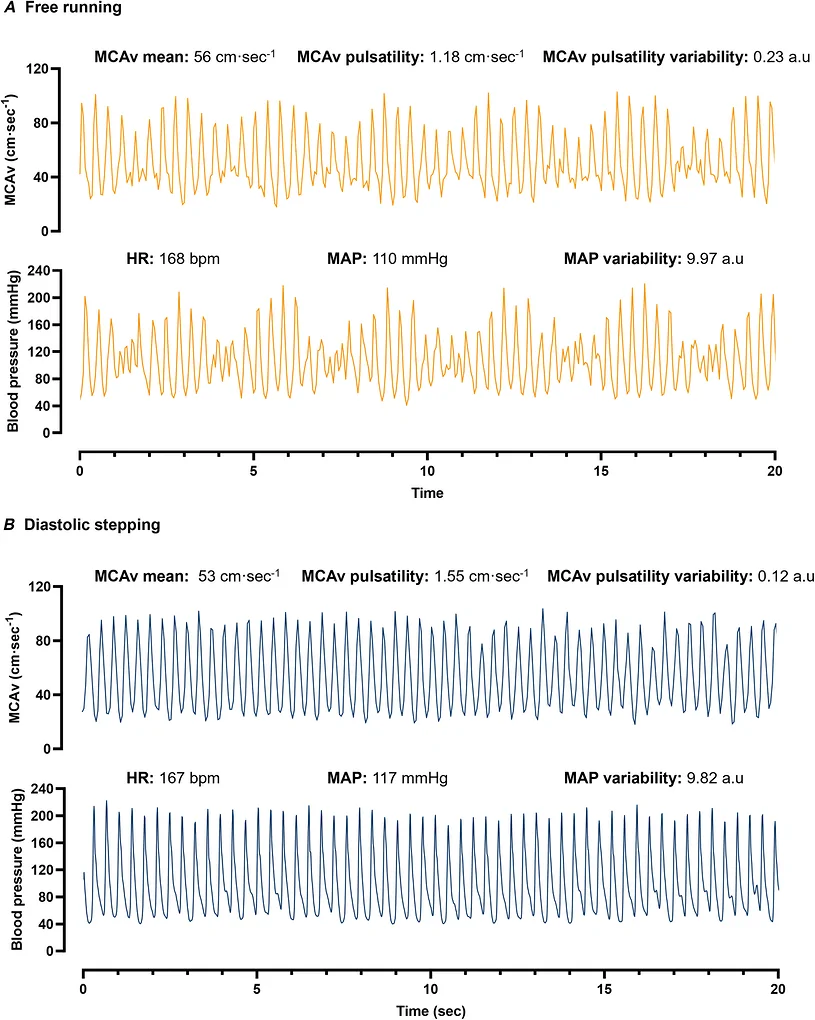
Example beat‐by‐beat middle cerebral artery velocity (MCA_v_) and blood pressure profiles during running Raw data illustrating the oscillating beat‐by‐beat blood pressure and MCA_v_ profile during free running, compared with stable profile when foot‐strike is synced to the diastolic phase of the cardiac cycle. Data presented are taken from a single participant, across the last 20 s of each condition. HR, heart rate; MAP, mean arterial pressure.

**Figure 5:**
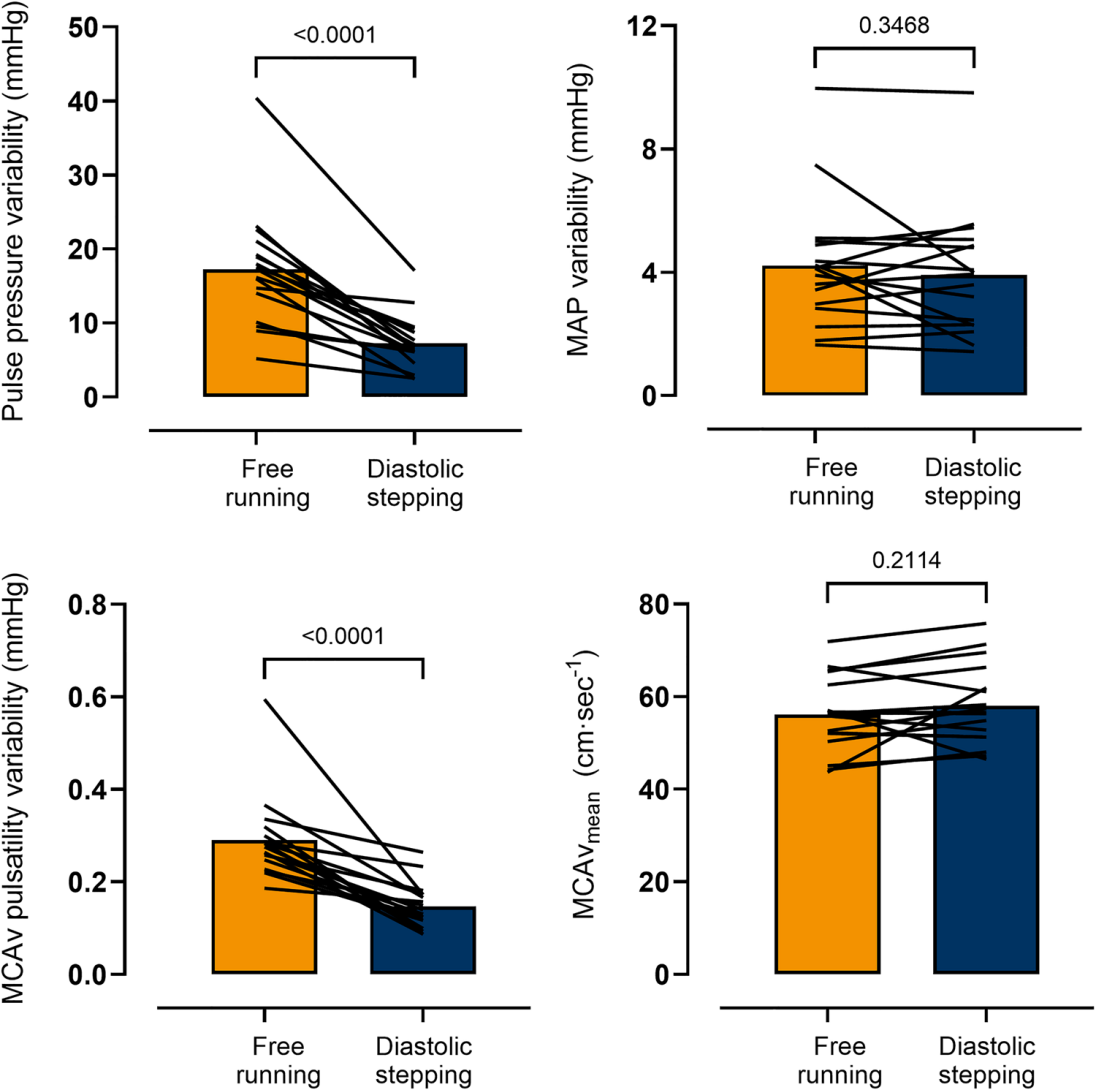
Pulsatility variability and middle cerebral artery velocity (MCA_v_) during manipulation of foot‐strike timing Beat‐by‐beat arterial and MCA_v_ pulsatility variability was reduced when foot‐strike was synced to diastole, compared with free running. However, mean arterial pressure (MAP) variability was similar between conditions. Despite reductions in pulsatility variability, MCA_v_ mean was not different between free running and diastolic stepping. Data are presented as means ± SD.

Participants also performed systolic stepping, although only five participants were able to consistently match their foot‐strike to the systolic phase of the cardiac cycle. Data across all three conditions in these five participants are shown in Table 4. Similar to diastolic stepping, pulse pressure variability and MCA_v_ pulsatility variability were reduced during systolic stepping, compared with free running, demonstrating a reduction in pulsatility oscillations. However, unlike diastolic stepping, average within‐beat pulse pressure and MCA_v_ pulsatility was lower during systolic stepping compared with free running and diastolic stepping. Despite marked differences in the pulsatility profile between conditions (i.e. Free running: pulsatility oscillations; Diastolic: reduced oscillations, high within‐beat pulsatility; Systolic: reduced oscillations, low within‐beat pulsatility), MCA_v_ mean was similar across all conditions.

**Table 4:** Cardiovascular and cerebrovascular haemodynamics during free running, diastolic and systolic stepping conditions

	Free running (*n* = 5)	Diastolic stepping (*n* = 5)	Systolic stepping (*n* = 5)	*Cohens d* (Free running *vs*. systolic stepping)	*Cohens d* (Diastolic stepping *vs*. systolic stepping)
*Cardiovascular haemodynamics*					
Heart rate (bpm)	163 ± 6	163 ± 6	166 ± 7	0.45	−0.49
MAP (mmHg)	91 ± 17	93 ± 17	89 ± 16	0.13	0.21
Systolic blood pressure (mmHg)	125 ± 32	135 ± 36	111 ± 29	0.47	0.73
Diastolic blood pressure (mmHg)	74 ± 11	73 ± 12	76 ± 12	0.15	−0.18
Pulse pressure (mmHg)	51.1 ± 25	61.2 ± 27.4	35.3 ± 37.6	0.68	**1.07**
MAP variability (mmHg)	3.0 ± 1.0	2.8 ± 1.5	2.4 ± 1.1	0.52	0.26
Systolic blood pressure variability (mmHg)	10.7 ± 2.9	5.4 ± 3.2	5.3 ± 2.3	**2.06**	0.06
Diastolic blood pressure variability (mmHg)	3.3 ± 1.5	3.2 ± 2.3	2.6 ± 1.5	0.49	0.34
Pulse pressure variability (mmHg)	12.4 ± 4.0	5.5 ± 2.8	6.9 ± 3.9	**1.38**	−0.42
*Cerebrovascular haemodynamics*					
MCA_v_ mean (cm s^−1^)	53.7 ± 9.0	53.9 ± 9.9	52.2 ± 10.2	0.15	0.17
MCA_v_ systolic (cm s^−1^)	96.5 ± 20.9	103.6 ± 23.2	89.4 ± 25.0	0.31	0.59
MCA_v_ diastolic (cm s^−1^)	28.3 ± 5,4	22.9 ± 2.2	29.3 ± 3.8	0.23	**−2.05**
MCA pulsatility (cm s^−1^)	1.26 ± 0.31	1.48 ± 0.17	1.12 ± 0.19	0.42	**1.29**
MCA pulsatility variability (cm s^−1^)	0.30 ± 0.06	0.18 ± 0.06	0.20 ± 0.05	**1.68**	−0.26
CVC (cm s^−1^ mmHg^−1^)	0.60 ± 0.13	0.58 ± 0.04	0.59 ± 0.08	0.09	0.09
$ET_{CO_{2}}$ (mmHg)	31.9 ± 2.7	30.4 ± 4.6	31.0 ± 3.4	0.28	−0.15

*Note*: Due to the small sample size, no statistical comparisons were performed between conditions. Instead, Cohen's *d* effect sizes are presented. Data are presented as means ± SD

Abbreviations: CVC, cerebrovascular conductance; $ET_{CO_{2}}$, End‐Tidal CO_2_; MAP, mean arterial pressure; MCA, middle cerebral artery; MCA_v_, middle cerebral artery velocity.

During the cognitive effort matched condition, pulse pressure variability and MCA_v_ pulsatility variability were not different from free running (*P* > 0.05, Table 5), demonstrating a similar oscillatory profile. Importantly, cardiovascular and cerebrovascular haemodynamics were not different between the free running and cognitive effort matched running condition (*P* > 0.05, Table 5), showing that matching foot‐strike to an auditory tone had no effect on cerebrovascular haemodynamics, independent of removing pulsatility oscillations.

**Table 5:** Cardiovascular and cerebrovascular haemodynamics during free running and cognitive effort matched conditions

	Free running (*n* = 11)	Cognitive effort matched (*n* = 11)	*P* value
*Cardiovascular haemodynamics*			
Heart rate (bpm)	162 ± 6	162 ± 6	0.613
MAP (mmHg)^†^	99.2 ± 18.8	99.8 ± 13.4	0.102
Systolic blood pressure (mmHg)^†^	139.0 ± 27.7	139.2 ± 33.6	0.957
Diastolic blood pressure (mmHg)^†^	73.2 ± 13.3	72.8 ± 13.5	0.605
Pulse pressure (mmHg)^†^	61.5 ± 30.9	64.7 ± 37.6	0.875
MAP variability (mmHg)^†^	4.8 ± 2.6	5.4 ± 6.2	0.664
Systolic blood pressure variability (mmHg)^†^	15.6 ± 8.2	15.8 ± 9.6	0.879
Diastolic blood pressure variability (mmHg)^†^	5.4 ± 2.8	6.1 ± 4.1	0.322
Pulse pressure variability (mmHg)^†^	18.3 ± 9.5	16.9 ± 7.5	0.564
*Cerebrovascular haemodynamics*			
MCA_v_ mean (cm s^−1^)	57.0 ± 8.9	57.6 ± 6.1	0.734
MCA_v_ systolic (cm s^−1^)	102.5 ± 17.8	103.0 ± 12.7	0.837
MCA_v_ diastolic (cm s^−1^)	29.8 ± 4.4	29.3 ± 5.3	0.759
MCA pulsatility (cm s^−1^)	1.29 ± 0.16	1.29 ± 0.19	0.997
MCA pulsatility variability (cm s^−1^)	0.29 ± 0.10	0.27 ± 0.04	0.505
CVC index (cm s^−1^ mmHg^−1^)	0.61 ± 0.17	0.58 ± 0.06	0.574
Ventilation (L min^−1^)	86.7 ± 11.5	88.0 ± 12.3	0.522
$ET_{CO_{2}}$ (mmHg)	31.6 ± 3.9	32.1 ± 5.3	0.555

*Note*: Statistical comparisons performed using paired *t* tests. Data are presented as means ± *SD*. †Comparisons between *n* = 9.

Abbreviations: CVC, cerebrovascular conductance; $ET_{CO_{2}}$, end‐tidal CO_2_; MAP, mean arterial pressure; MCA, middle cerebral artery; MCA_v_, middle cerebral artery velocity.

#### Protocol 3 – Cerebrovascular CO_2_ reactivity

The changes in cerebrovascular and ventilatory parameters during assessment of CO_2_ reactivity during running are shown in Table 6. Due to insufficient increases in $ET_{CO_{2}}$ in two participants, reactivity to hypercapnia is only presented for *n* = 10, but reactivity to hypocapnia is presented for *n* = 12. During the hypercapnia trial, the increase in $ET_{CO_{2}}$ was not different between rest and exercise (*P* = 0.41), but the increase in MCA_v_ mean was numerically greater during exercise, although not significantly (*P* = 0.67). Consequently, a small and significant increase in CVR index to hypercapnia was observed from rest to exercise (*P = *0.049, Fig. 6). However, when represented as relative change there was no difference in CVR index from hypercapnia (*P = *0.32) (Table 6). During the hypocapnia trial, the decrease in $ET_{CO_{2}}$ was not different during exercise, compared with rest (*P* = 0.96). However, the decrease in MCA_v_ mean was markedly attenuated (*P* < 0.0001) and almost abolished (Rest: Δ−12.2 ± 6.3 cm s^−1^ *vs*. Running Exercise: Δ−1.2 ± 3.1 cm s^−1^). This attenuation resulted in a large decrease in CVR index to hypocapnia from rest to exercise (*P* = 0.006), regardless of whether it was represented as absolute or relative change (*P* < 0.0001; Table 6).

**Table 6:** Changes in cerebrovascular haemodynamics during assessment of CO_2_ reactivity during running exercise (Protocol 3)

	Hypercapnia trial (*n* = 10)		*P* value	Hypocapnia trial (*n* = 12)		*P* value
	Rest	Running exercise		Rest	Running exercise	
MCA_v_ (cm s^−1^)	Δ11.8 ± 5.2	Δ12.7 ± 5.4	0.67	Δ−12.2 ± 6.3	Δ−1.2 ± 3.1	**<0.0001**
$ET_{CO_{2}}$ (mmHg)	Δ9.6 ± 4.2	Δ8.5 ± 3.6	0.41	Δ−11.5 ± 5.0	Δ−11.6 ± 4.3	0.96
CVR index (cm s^−1^ mmHg^−1^)	1.26 ± 0.29	1.59 ± 0.54	**0.049**	−0.79 ± 0.44	−0.19 ± 0.50	**0.006**
CVR index (%/mmHg)	2.60 ± 0.56	2.93 ± 1.1	0.32	−1.90 ± 0.66	−0.17 ± 0.52	**<0.0001**

*Note*: Statistical comparisons were performed between rest and exercise using paired *t* tests. Data are presented as means ± SD.

Abbreviations: CVR, cerebrovascular reactivity; $ET_{CO_{2}}$, end‐tidal CO_2_; MCA_v_, middle cerebral artery velocity.

**Figure 6:**
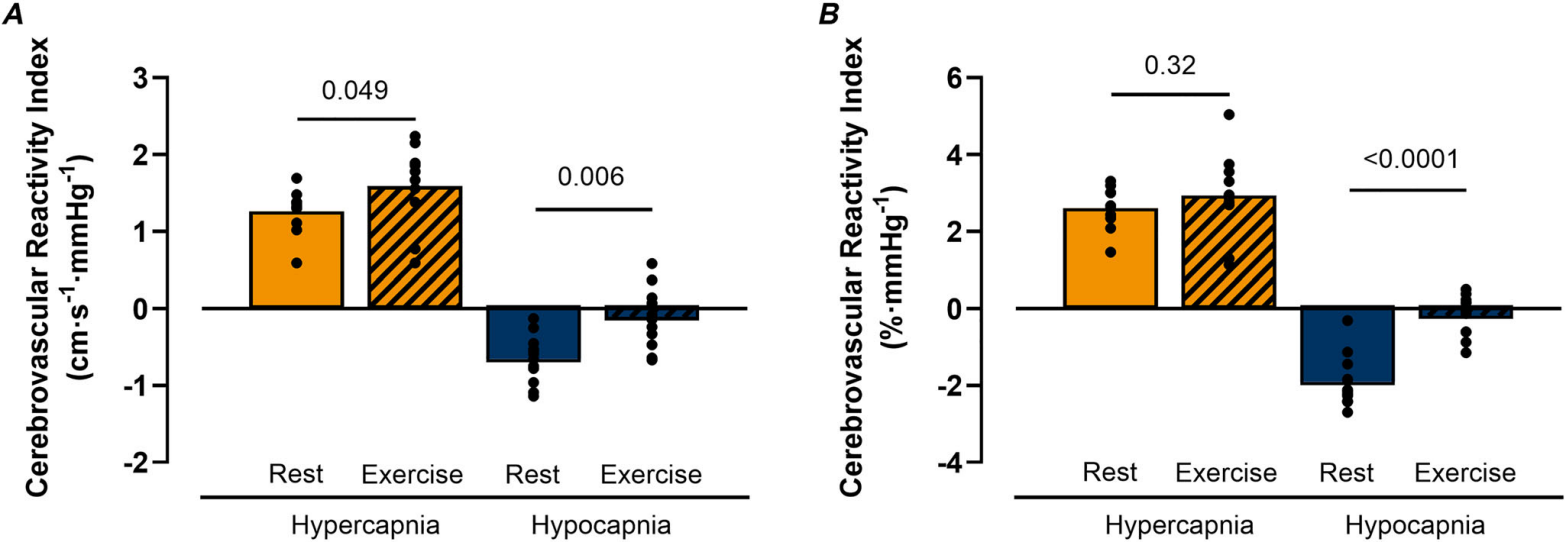
Cerebrovascular CO_2_ reactivity at rest and during running exercise (Protocol 3) expressed as absolute change (*A*) and relative change (*B*) Middle cerebral artery velocity (MCA_v_) reactivity to hypercapnia (*n* = 10) is increased during running exercise, *versus* rest, but MCA_v_ reactivity to hypocapnia (*n* = 12) is reduced during running. *P < 0.05. Cerebrovascular reactivity index calculated as the ratio of ΔMCA_v_ or % ΔMCA_v_ divided by Δ$ET_{CO_{2}}$. Statistical comparisons performed using paired *t* tests between rest and exercise. ns = non‐significant. Data are presented as means ± SD.

### Study 2

#### Protocol 4 – Cerebrovascular responsiveness to hypocapnia during running and cycling at matched BP

Cerebrovascular responsiveness to hypocapnia was assessed at rest and during running and cycling with MAP matched between modalities. As expected, there was an effect of condition on MAP (ANOVA: *P* = 0.013), with MAP increasing above rest (94 ± 12 mmHg) during both cycling (106 ± 17mmHg, *P* = 0.023) and running exercise (107 ± 14 mmHg, *P* = 0.056). However, by design, there were no differences in MAP between running and cycling (*P* = 0.937). Change in $ET_{CO_{2}}$ (∆$ET_{CO_{2}}$) during the cerebrovascular reactivity tests did not differ across conditions (Rest: −13.9 ± 3.3 mmHg; Running: −14.7 ± 2.9 mmHg; Cycling: 13.0 ± 2.2 mmHg, ANOVA *P* = 0.438). However, the change in MCA_v_ (∆MCA_v_) did differ across conditions (Rest: −16.2 ± 7.2 cm s^−1^; Running: −9.6 ± 5.4 cm s^−1^; Cycling: −9.5 ± 6.3 cm s^−1^, ANOVA *P* = 0.003). MCA_v_ reactivity to a given $ET_{CO_{2}}$ was altered from rest to exercise (Fig. 8), but not different between modalities (Fig. 8). Consequently, CVR index was also different across conditions (ANOVA *P* = 0.003, Fig. 8), where CVR was reduced below rest (−1.21±0.59 cm s^−1^ mmHg^−1^) during both cycling (−0.72 ± 0.49 cm s^−1^ mmHg^−^1, *P* = 0.01) and running exercise (−0.72±0.49 cm s^−1^ mmHg^−^1, *P* = 0.006), with no difference in CVR between exercise modalities (*P* = 0.999, Fig. 8). These results indicate that MCA_v_ responsiveness is reduced during exercise, but comparable during exercise modalities at similar levels of MAP.

#### Protocol 5 – Effect of exercise intensity and BP on cerebrovascular responsiveness to hypocapnia

Cerebrovascular responsiveness to hypocapnia was assessed at rest and during three steady‐state exercise workloads (64 ± 18, 105 ± 8.4 and 155 ± 36 W). As expected, there was an effect of condition on MAP (Rest: 94 ± 7 mmHg; 64 W: 101 ± 13 mmHg; 105 W: 109 ± 16 mmHg; 155 W: 113 ± 17 mmHg, ANOVA: *P* = 0.011), where MAP increased progressively, above rest, with increasing exercise intensity. Change in $ET_{CO_{2}}$ (∆$ET_{CO_{2}}$) during the cerebrovascular reactivity tests did not differ across conditions (Rest: −14.5 ± 2.5 mmHg; 50 W: −17.1 ± 2.6 mmHg; 100 W: −14.9 ± 2.2 mmHg; 150 W: −12.6 ± 2.0 mmHg, ANOVA *P* = 0.140). However, the change in MCA_v_ (∆MCA_v_) was different across conditions (Rest: −15.1 ± 7.4 cm s^−1^; 50 W: −19.4 ± 8.2 cm s^−1^; 100 W: −11.5 ± 4.7 cm s^−1^; 150 W: −4.1 ± 2.2 cm s^−1^ ANOVA *P* = 0.021), showing that MCA_v_ reactivity to a given $ET_{CO_{2}}$ was altered with exercise. Consequently, CVR index (∆MCA_v_/∆(∆$ET_{CO_{2}}$) showed no difference from rest at 64 W (*P* = 0.239, Fig. 7) but progressively decrease below resting values at 105 W (*P* = 0.044, Fig. 7), with a further decline from 105 to 155 W (*P* = 0.057, Fig. 7). Additionally, there was a significant negative relationship between ∆MAP and ∆CVR index during exercise (Slope = −0.043, *R* ^2 = ^0.62, *P* < 0.0001), indicating that for every 1 mmHg increase in MAP there was a 0.04 cm s^−1^ mmHg^−1^ reduction in MCA_v_ reactivity.

**Figure 7:**
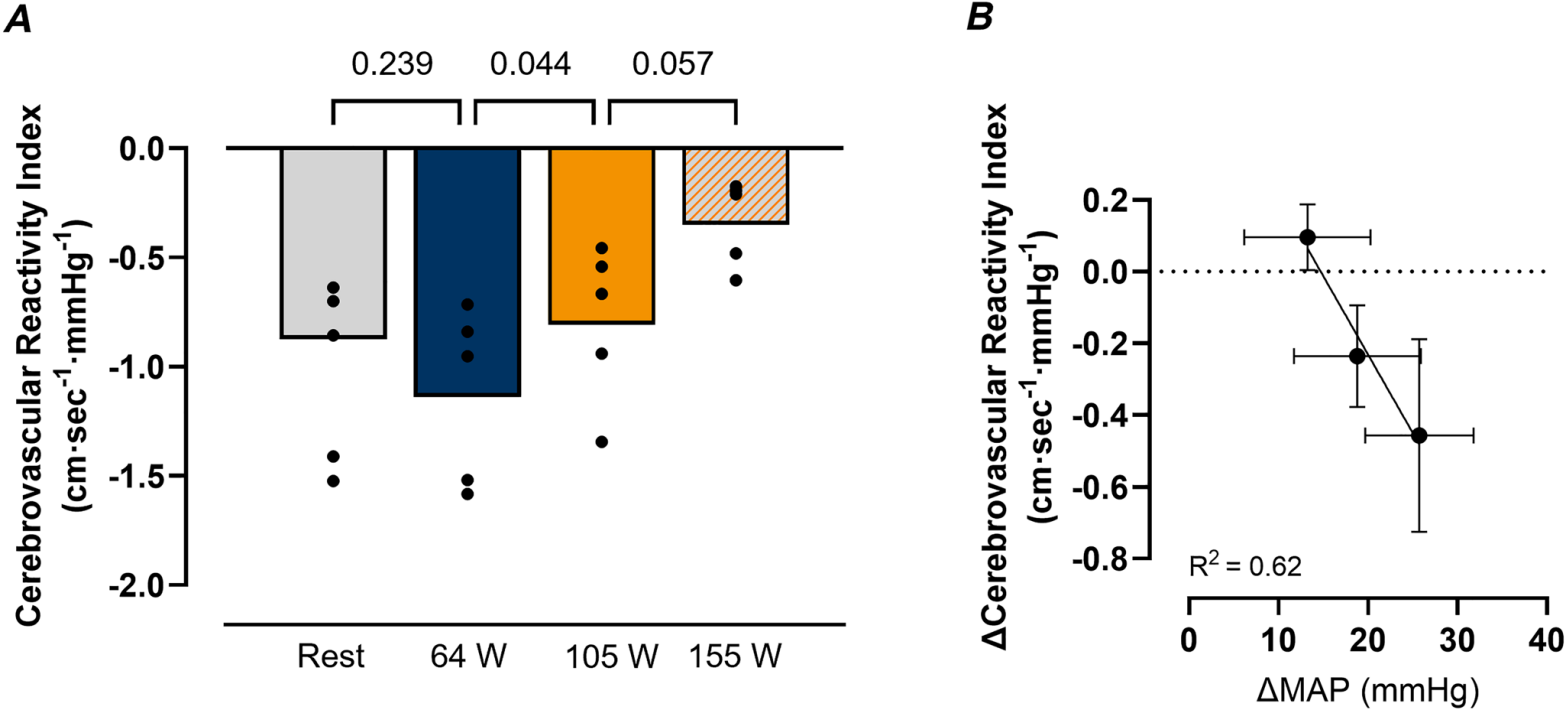
Effect of exercise intensity and prevailing blood pressure on cerebrovascular reactivity (*A*) and relationship between absolute change in middle cerebral artery velocity (MCA_v_) CO_2_ reactivity and mean arterial pressure (MAP) during cycling exercise (*B*) MCA_v_ reactivity to hypocapnia (*n* = 5) decreases progressively with increasing cycling exercise intensity and the change in MCA_v_ reacivity during exercise was related to change in MAP. Cerebrovascular reactivity index calculated as the ratio of ΔMCA_v_ divided by Δ$ET_{CO_{2}}$_._ Statistical comparisons performed using one‐way repeated measures ANOVA and linear regression. Data are presented as means ± SD.

**Figure 8:**
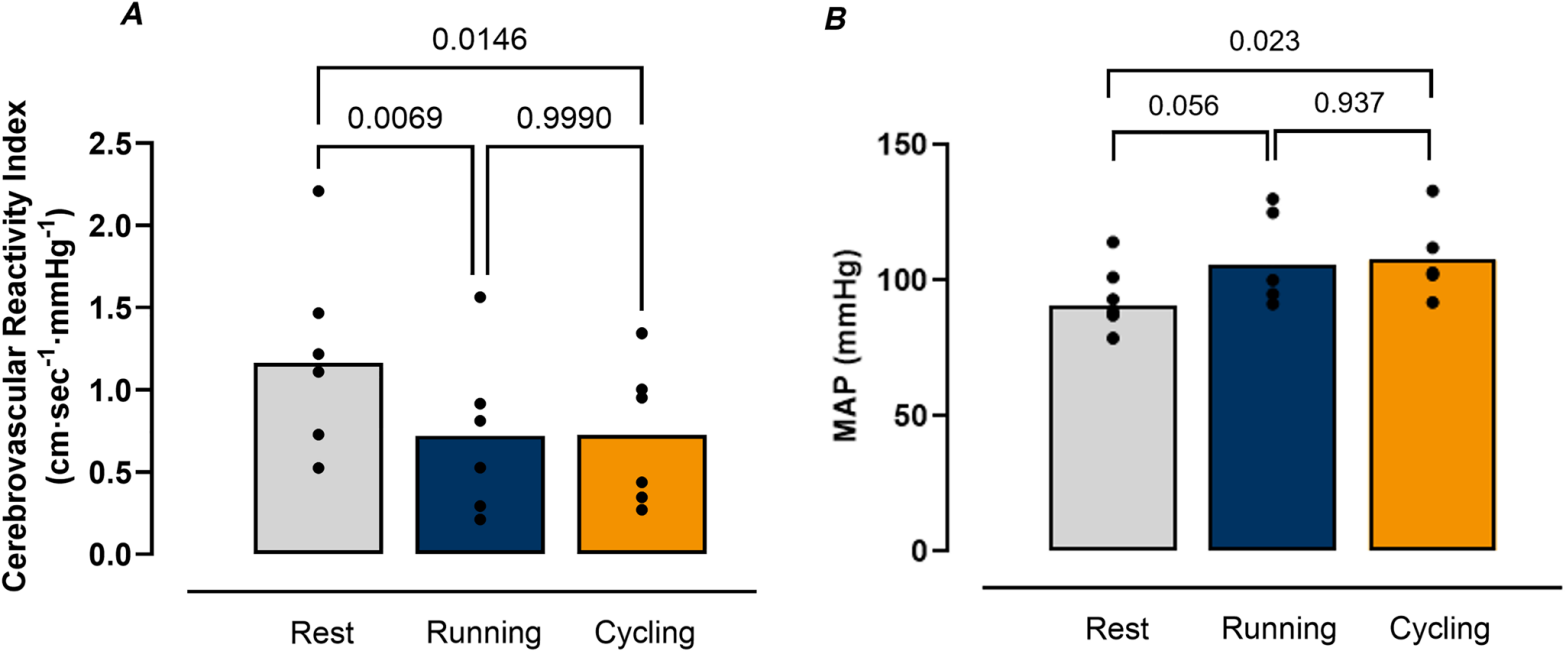
(*A*) Cerebrovascular reactivity and (B) mean arterial pressure (MAP) during cycling and running exercise when BP matched between modalities AMiddle cerebral artery velocity (MCA_v_) reactivity to hypocapnia (*n* = 6) decreased below rest during exercise, with no difference between cycling and running when modalities matched for MAP. Cerebrovascular reactivity index calculated as the ratio of ΔMCA_v_ divided by Δ$ET_{CO_{2}}$_._ Statistical comparisons performed using one‐way repeated measures ANOVA. Data are presented as means ± SD.

## Discussion

The aim of the present study was to examine the mechanisms regulating MCA_v_ during running exercise, specifically the influence of pulsatility oscillations and cerebrovascular reactivity to CO_2_. The primary findings of Study 1 were: (1) MCA_v_ increases linearly during incremental treadmill running in both males and females, despite decreases in $ET_{CO_{2}}$ at higher exercise intensities (80% and 100% ${\dot{V}}_{O_{2}\max}$); (2) synchronizing foot‐strike to either the diastolic or systolic phase of the cardiac cycle removed the oscillations in arterial and MCA_v_ pulsatility but did not influence MCA_v_ mean responses; and (3) MCA_v_ reactivity to hypercapnia was increased during running but MCA_v_ reactivity to hypocapnia was reduced by approximately 75%. The primary findings of Study 2 were: (1) MCA_v_ reactivity to hypocapnia decreased progressively with increasing exercise intensity during cycling and was related to the increase in MAP; (2) MCA_v_ reactivity to hypocapnia was similar between cycling and running when increases in MAP were matched between modalities. Collectively, these findings suggest that the progressive increase in MCA_v_ during running exercise is due, at least in part, to an attenuated cerebrovascular responsiveness to hypocapnia. Furthermore, the reduction in MCA_v_ reactivity to hypocapnia appears to be related to the prevailing BP and thus likely reflects the magnitude of static autoregulation and vasoconstrictor capacity rather than an effect of modality or intrinsic sensitivity to CO_2_ *per se*.

### MCA_v_ responses to treadmill running

CBF responses to exercise typically show a biphasic response, increasing up to 60% of ${\dot{V}}_{O_{2}\max}$ before declining at higher intensities (Furlong et al. 2020; Moraine et al. 1993; Smith & Ainslie 2017). Most research has focused on cycling exercise, but more recent studies reveal apparent differences across exercise modalities (Faull et al. 2015; Furlong et al. 2020). For example, no decrease in MCA_v_ mean is observed during high‐intensity rowing (30 s all‐out effort; Faull et al., 2015) and MCA_v_ mean progressively increases during incremental running exercise, albeit only in ‘high fit’ individuals (Furlong et al. 2020). Our findings also demonstrate a non‐typical response to running, with MCA_v_ mean increasing progressively and peaking at maximal exercise in both males and females. These findings provide further evidence of apparent modality‐dependent responses and highlight potential differences in regulatory control of CBF across exercise modalities.

### Influence of pulsatility oscillations on MCA_v_ responses to running

Previous studies have speculated that cyclic oscillations in arterial pulsatility observed during running may influence CBF regulation and explain divergent responses with running (Furlong et al., 2020; Lyngerra et al., 2013). These distinctive oscillations, observed by ourselves and previously by others (i.e. beat phenomenon (Furlong et al. 2020; Leahy et al., 2024; Lyngeraa et al. 2013; Palatini et al., 1989)), are thought to be mediated by the interaction between the forward travelling, cardiac‐generated pressure wave and a backward travelling, inertia pressure wave generated by the mechanical impact of the foot‐strike (Palatini et al. 1989), which influence cardiac afterload and stroke volume (Wakeham et al. 2023). While it has been reported that the frequency of running‐induced arterial and MCA_v_ pulsatility oscillations are related to the difference in heart rate and stride frequency (Lyngeraa et al. 2013; Palatini et al. 1989), the effect of experimentally manipulating foot‐strike timing on these oscillations had not been previously examined. In the present study, timing the foot‐strike to either the diastolic or systolic phase of the cardiac cycle, using step‐guidance Counterpace technology, successfully removed these oscillations. Thus, we demonstrate that beat‐to‐beat pulsatility oscillations with running are mediated by variations in the timing of the foot‐strike relative to the cardiac cycle, and presumably the timing of the backward travelling pressure wave.

Importantly, these foot strike‐induced oscillations are associated with large beat‐to‐beat variability in systolic and diastolic BP (Palatini et al. 1989), and it has been proposed that this challenges cerebral autoregulatory mechanisms. Dynamic cerebral autoregulation, which usually dampens the effects of BP changes on CBF, is likened to a high‐pass filter that effectively buffers slow BP changes but becomes progressively less effective as the frequency of BP changes increases (Hamner et al., 2004, 2019; Tan 2012; Zhang et al. 1998). The high‐frequency BP oscillations during running likely occur too fast to be counteracted (Lyngerra et al., 2013), meaning that they are directly transferred to the cerebral circulation, resulting in no effective autoregulation. As a result, MCA_v_ may more passively follow BP changes, overriding CO_2_‐induced vasoconstriction_._ If foot strike‐induced oscillations impair cerebral autoregulation during running, then removing these oscillations would reverse any impairment and any associated pressure‐driven increases in CBF. However, contrary to the hypothesis, removing these oscillations had no effect on MCA_v_ mean responses, suggesting that foot strike‐induced pulsatility oscillations do not cause the cerebral circulation to become more pressure‐passive during running.

During free running, systolic and diastolic BP show marked variability, which was reduced during diastolic and systolic stepping. In contrast, variability in MAP was remarkably similar across all conditions. Thus, the oscillations in systolic and diastolic BP appear to be similar in magnitude but opposite in direction, resulting in relatively stable MAP between beats. This may explain why the oscillatory flow profile had no effect on mean MCA_v,_ as fluctuations in cerebral perfusion pressure were small, because MAP remained relatively constant. Thus, the autoregulatory challenge imposed by these oscillations appears limited and likely explains why removing them did not alter mean MCA_v_. In line with this, Cornwell and colleagues (Cornwell et al. 2014) reported minor effects of reduced pulsatility on MCA_v_ mean and cerebral autoregulation in patients with left ventricular assist devices. Overall, our results demonstrate that oscillations in pulsatility during running do not appear to affect MCA_v_ mean and are not a primary mechanism explaining why MCA_v_ mean continues to increase at higher exercise intensities with running.

### Cerebrovascular CO_2_ reactivity during running

Arterial carbon dioxide is a major regulator of CBF via its effect on cerebrovascular tone. Hypocapnia elicits cerebral vasoconstriction and reductions in CBF, whereas hypercapnia elicits cerebral vasodilatation and increases in CBF. During cycling exercise, MCA_v_ is closely coupled to $ET_{CO_{2}}$ (Moraine et al. 1993). In contrast, we observed an uncoupling of MCA_v_ mean and $ET_{CO_{2}}$ during high‐intensity running exercise, where MCA_v_ mean increased at 60% ${\dot{V}}_{O_{2}\max}$, 80% ${\dot{V}}_{O_{2}\max}$ and maximal exercise, despite progressive reductions in $ET_{CO_{2}}$. One possible explanation is a reduced cerebrovascular responsiveness to hypocapnia, where a given reduction in $ET_{CO_{2}}$ elicits less cerebral vasoconstriction. In the present study, we observed an ∼25% increase in CVR to hypercapnia during high‐intensity running, but a marked blunting of CVR to hypocapnia (∼75% reduction). In fact, the decrease in MCA_v_ mean during reductions in $ET_{CO_{2}}$ was almost abolished. Consequently, the attenuation in CVR to hypocapnia appears to be a primary mechanism explaining the absence of the characteristic decline in MCA_v_ at higher exercise intensities with running. The enhanced hypercapnic responsiveness is consistent with previous studies reporting an increase in MCA_v_ reactivity to hypercapnia during cycling exercise (Murrell et al. 2013; Ogoh et al. 2008). In contrast, only two studies have examined MCA_v_ reactivity to hypocapnia during exercise (Ogoh et al., 2008; Rasmussen et al., 2006). Ogoh et al. (2008) reported no difference in hypocapnic reactivity during low‐intensity cycling (32 ± 2% ${\dot{V}}_{O_{2}\max}$). However, these findings are not representative of cerebrovascular regulation at higher intensities where increases in BP and hypocapnia become more pronounced. Rasmussen et al. (2006), who studied participants during moderate‐intensity cycling (∼67% ${\dot{V}}_{O_{2}\max}$), reported an increased cerebrovascular CO_2_ reactivity. However, Rasmussen & colleagues (2006) derived a single MCA_v_ CO_2_ reactivity curve by integrating the hypercapnic and hypocapnic responses using a double‐logarithmic regression analysis. While the combined analysis suggested an improved cerebrovascular CO_2_ reactivity, visual inspection of the data indicates increased hypercapnic reactivity but decreased hypocapnic reactivity. This ‘hysteresis’ in the regulation of hypercapnic and hypocapnic responsiveness during exercise also likely explains their observation that the CO_2_ reactivity relationship changed from linear at rest to curvilinear during exercise.

Our data are in line with both findings including no change in hypocapnic reactivity at low‐intensity exercise (Fig. 7*A*), an improved hypercapnic reactivity during more intense exercise (Fig. 6*A*) and a reduction in cerebrovascular responsiveness to hypocapnia during more intense exercise. Interestingly, this likely does not represent an impaired intrinsic vascular sensitivity to CO_2_ *per se*. Instead, we speculate that the blunted responsiveness to hypocapnia reflects a reduced cerebrovascular vasoconstrictor reserve. Elevations in BP during moderate‐ to severe‐intensity exercise increase cerebral perfusion pressure and evoke autoregulatory vasoconstriction to maintain stable CBF. As exercise intensity and BP increase further, this autoregulatory vasoconstriction would progressively increase, elevating cerebrovascular tone and shifting the cerebral vessels closer to their vasoconstrictor limit. Under these conditions, the capacity to vasoconstrict in response to hypocapnia, or any other vasoconstrictor stimulus, may be reduced, attenuating the cerebrovascular response. This hypothesis would also explain the increased responsiveness to hypercapnia during running, as pre‐constricted vessels with a high vascular tone would have a greater dilatory capacity. Furthermore, it would also explain the lack of difference in hypocapnic reactivity observed by Ogoh and colleagues at low‐intensity exercise, as this was an exercise intensity with no significant increase in MAP, and thus no significant autoregulatory vasoconstriction (Ogoh et al., 2008). Our findings from Study 2 are consistent with this interpretation, demonstrating that progressive increases in BP with increasing exercise intensity are accompanied by progressive reductions in cerebrovascular responsiveness to hypocapnia. Although these findings do not establish causality, they suggest that the prevailing BP may be an important determinant of cerebrovascular CO_2_ responsiveness during exercise. Indeed, progressive reductions in BP, where autoregulation would *reduce* cerebrovascular tone, has been shown to elicit marked *reductions* in CVR to hypercapnia (Harper & Glass 1965; Iwabuchi et al. 1973), presumably by reducing available dilatory reserve.

Why reduced cerebrovascular CO_2_ reactivity appears to be more evident during running than cycling is unclear. However, we speculate that this may be explained by higher absolute oxygen uptake, cardiac output and BP typically achieved during running *versus* cycling (Leahy et al. 2024), due to greater muscle mass engagement (Leahy et al. 2024; Millet et al., 2009). Thus, running is more likely to recruit a greater proportion of the available cerebral vasoconstrictor reserve to offset greater increases in cerebral perfusion pressure, rather than an intrinsic effect of exercise modality. Indeed, in the current study, the cerebral vasoconstrictor response to CO_2_ is similar between running and cycling if the rise in BP is matched between modalities. If our hypothesis is correct, we would also expect fitter individuals who achieve higher peak absolute oxygen uptakes and thus cardiac outputs and BP to have a more blunted response to hypocapnia during exercise. Indeed, an important finding from Furlong and colleagues is that MCA_v_ increases linearly during running in ‘high fit’ individuals (male: > 45 mL min^−1^ kg^−1^, female: >40 mL min^−1^ kg^−1^), whereas ‘low fit’ individuals (male: < 40 mL min^−1^ kg^−1^, female: < 35 mL min^−1^ kg^−1^) exhibited the typical decrease in MCA_v_ mean at higher running intensities. In line with this, participants in the present study were well‐trained (Average ${\dot{V}}_{O_{2}\max}$: 58.5 ± 10.2 mL min^−1^ kg^−1^) and notably, only one participant, with the lowest cardiorespiratory fitness (${\dot{V}}_{O_{2}\max}$: 36 mL min^−1^ kg^−1^) exhibited a return of MCA_v_ mean to baseline values at maximal exercise.

### Perspective

During low‐intensity exercise, the increase in CBF is primarily driven by neurovascular coupling, where increases in neuronal activation produce regional metabolic vasodilatation. As exercise intensity and MAP progressively increase, this regional metabolic vasodilatation occurs alongside an autoregulatory vasoconstriction (i.e. static autoregulation) within the non‐metabolically active regions of the brain, to offset the rise in cerebral perfusion pressure. Independent of modality, at low absolute exercise intensities (i.e. lower oxygen uptake) the degree of autoregulatory vasoconstriction will be modest to offset only a modest rise in BP. This preserves the ability of reductions in arterial CO_2_ to decrease CBF, even during relatively intense exercise in ‘lower fit’ individuals. Alternatively, in highly fit individuals with high absolute oxygen uptakes, we speculate that autoregulatory vasoconstriction reaches its maximal capacity during relatively moderate to intense exercise, due to greater increases in BP. Beyond this point, the cerebral circulation becomes pressure‐passive and CBF continues to rise with exercise intensity. It is important to state that in these individuals it is hypothesized that the cerebral circulation will be unresponsive to *any* vasoconstrictor stimuli, not only CO_2_. Thus, we propose that the reduced hypocapnic responsiveness does not reflect a decrease in intrinsic CO_2_ sensitivity *per se*, but rather autoregulation pushing the cerebral circulation further towards its maximal vasoconstrictor capacity.

### Methodological considerations

First, TCD was used to assess CBF, which only quantifies blood velocity through the MCA, not flow. TCD assumes a constant diameter for changes in velocity to reflect changes in flow. However, altered larger intra‐cranial artery diameter has been reported during changes in arterial blood gases, BP and exercise (Ainslie & Hoiland 2014; Coverdale et al. 2014; Giller et al. 1993; Wilson et al. 2011). Yet, despite its limitations, TCD remains the only method allowing quantification of CBF during running exercise. Moreover, TCD would serve to underestimate the magnitude of change in flow during alterations in CO_2_, which would not alter the interpretation of our results (i.e. cerebrovascular CO_2_ reactivity to hypocapnia is reduced during exercise). Second, we did not assess BP during cerebrovascular CO_2_ reactivity, which would allow quantification of the relationship between changes in cerebrovascular conductance/resistance and $ET_{CO_{2}}$. Increases in CBF during hypercapnia are not exclusively mediated by vascular reactivity but partly mediated by the CO_2_‐induced pressor response (Regan et al. 2014). Thus, it is possible that the hypercapnia‐induced pressor response was greater during running exercise *versus* rest, which would have increased our measure of CO_2_ reactivity, independent of any change in vessel reactivity. However, even extreme hypocapnia does not appreciably change BP (Willie et al. 2012); thus, it would not affect the interpretation of our primary finding (i.e. cerebrovascular CO_2_ reactivity to hypocapnia is reduced during exercise). Third, $ET_{CO_{2}}$ was used as a surrogate for $P_{\mathrm{aC}O_{2}}$. The end‐tidal to arterial $P_{CO_{2}}$ difference may widen during exercise with increases in tidal volume and metabolic CO_2_ production (Jones et al., 1979; Robbins et al., 1990). This could have led to an overestimation of the change in $P_{\mathrm{aC}O_{2}}$ during exercise and affected our estimation of CVR. To account for this, we estimated arterial $P_{CO_{2}}$ using a validated corrective equation (Jones et al., 1979) and calculated CVR. Regardless of whether $ET_{CO_{2}}$ or estimated $P_{\mathrm{aC}O_{2}}$ was used, cerebrovascular reactivity to hypocapnia was reduced during running exercise (∆MCA_v_/∆$ET_{CO_{2}}$; Rest: 1.00 ± 0.27 cm s^−1^ mmHg^−1^, Running: 0.32 ± 0.24 cm s^−1^ mmHg^−1^; ∆MCA_v_/∆Estimated $P_{\mathrm{aC}O_{2}}$; Rest: 1.08 ± 0.30 cm s^−1^mmHg^−1^, Running: 0.37 ± 0.27 cm s^−1^ mmHg^−1^). Fourth, matching foot‐strike to a metronome increases cognitive effort, potentially increasing blood flow to specific brain regions, via neurovascular coupling. Such increases could have masked the hypothesized decrease in MCA_v_ mean resulting from the removal of pulsatility oscillations alone. To account for this, a subset of participants matched their foot‐strike to a randomly generated auditory tone, to match the pulsatility profile to that of free running but the cognitive effort and neuronal activation to the diastolic stepping condition. Importantly, we show no differences in any cardiovascular or cerebrovascular haemodynamics between free running and cognitively matched condition. Thus, MCA_v_ mean did not appear to be independently affected by matching foot‐strike to metronome *per se*. Fifth, while arterial and MCA_v_ pulsatility variability (i.e. beat‐to‐beat pulsatility) was reduced during diastolic stepping, average within‐beat pulsatility increased. Increased pulsatile pressure and/or blood flow may increase cerebral shear stress‐mediated dilatation, leading to an increase in CBF. This increase could have masked the hypothesized reduction in MCA_v_ mean (Zhang & Levine 2007) with removal of pulsatility oscillations. However, in a subset of participants, BP and MCA_v_ mean was also comparable during systolic stepping, where pulse pressure and average within‐beat MCA pulsatility was reduced. These findings suggest that altered within‐beat pulsatility has no major effect on MCA_v_ mean responses.

### Conclusion

Continued increases in regional cerebral perfusion during incremental running exercise, despite progressive reductions in $ET_{CO_{2}}$, are due to reduced cerebrovascular responsiveness to hypocapnia and not influenced by foot strike‐induced oscillations in pulsatility. Reductions in cerebrovascular responsiveness during running may be related to progressive autoregulatory vasoconstriction with increasing BP, which approaches cerebrovascular vasoconstrictor reserve, where little additional constriction is available despite further hypocapnia.

## Additional information

## Competing interests

None declared.

## Author contributions

J.S.L. and L.L.S. conceived and designed the study. J.S.L., L.L.S., D.R.P., C.A.H. and C.D.B. performed experiments. J.S.L., L.L.S., D.R.P., C.A.H. and W.K.C. contributed to the analysis and interpretation of the data. J.S.L. and L.L.S. drafted the manuscript, and all authors revised it critically for important intellectual content. All authors approved the final version of the manuscript and agree to be accountable for all aspects of the work. All persons designated as authors qualify for authorship, and all those who qualify for authorship are listed.

## Funding

No funding was received to support this work.

## Generative AI statement

No generative AI tools were used in the preparation of this manuscript.

## Supporting information


Peer Review History

## Data Availability

Data are available upon reasonable request.
