# Supplementary material for: Mechanisms regulating cerebral perfusion during running exercise: Influence of foot strike‐induced pulsatility oscillations, CO2 reactivity and BP
Source: J Physiol. 2026 Sep 1;604(20):8501–22. doi: 10.1113/JP288117 (PMC13674218; doi:10.1113/JP288117)
Supplement: Supplementary file 1 [file TJP-604-8501-s001.pdf]

The Journal of Physiology

Mechanisms regulating cerebral perfusion during running: Influence of foot-strike induced pulsatility oscillations, CO2 reactivity and blood pressure

Lydia L Simpson, Dean R Perkins, Connor A Howe, Christina Dominique Bruce, William K Cornwell III, and Justin Stevan Lawley
DOI: 10.1113/JP288117

Corresponding author(s): Lydia Simpson (Lydia.simpson@bristol.ac.uk)

The following individual(s) involved in review of this submission have agreed to reveal their identity: Shigehiko Ogoh (Referee #1)

Review Timeline:

Submission Date:	15-Nov-2024
Editorial Decision:	14-Jan-2025
Revision Received:	07-Aug-2026
Editorial Decision:	12-Aug-2026
Revision Received:	13-Aug-2026
Accepted:	17-Aug-2026

Senior Editor: Laura Bennet

Reviewing Editor: Philip Ainslie

Transaction Report:

(Note: With the exception of the correction of typographical or spelling errors that could be a source of ambiguity, letters and reports are not edited. Depending on transfer agreements, referee reports obtained elsewhere may or may not be included in this compilation. Referee reports are anonymous unless the Referee chooses to sign their reports.)

Dear Dr Simpson,

Re: JP-RP-2024-288117 "Mechanisms regulating cerebral perfusion during running exercise: Influence of foot-strike induced pulsatility oscillations and CO2 reactivity" by Lydia L Simpson, Dean R Perkins, William K Cornwell III, and Justin Stevan Lawley

Thank you for submitting your manuscript to The Journal of Physiology. It has been assessed by a Reviewing Editor and by 1 expert referee and we are pleased to tell you that it is potentially acceptable for publication following satisfactory major revision.

Please advise your co-authors of this decision as soon as possible.

The referee reports are copied at the end of this email.

Please address all the points raised and incorporate all requested revisions or explain in your Response to Referees why a change has not been made. We hope you will find the comments helpful and that you will be able to return your revised manuscript within 9 months. If you require longer than this, please contact journal staff: jp@physoc.org. Please note that this letter does not constitute a guarantee for acceptance of your revised manuscript.

Your revised manuscript should be submitted online using the link in your Author Tasks: Link Not Available. This link is accessible via your account as Corresponding Author; it is not available to your co-authors. If this presents a problem, please contact journal staff (jp@physoc.org). Image files from the previous version are retained on the system. Please ensure you replace or remove any files that are being revised.

If you do not wish to submit a revised version of your manuscript, you must inform our journal staff (jp@physoc.org) or reply to this email to request withdrawal. Please note that a manuscript must be formally withdrawn from the peer review process at one journal before it may be submitted to another journal.

TRANSPARENT PEER REVIEW POLICY: To improve the transparency of its peer review process, The Journal of Physiology publishes online as supporting information the peer review history of all articles accepted for publication. Readers will have access to decision letters, including Editors' comments and referee reports, for each version of the manuscript, as well as any author responses to peer review comments. Referees can decide whether or not they wish to be named on the peer review history document.

ABSTRACT FIGURES: Authors are expected to use The Journal's premium BioRender account to create/redraw their Abstract Figures. Information on how to access this account is here: https://physoc.onlinelibrary.wiley.com/journal/14697793/biorender-access.

This will enable Authors to create and download high-resolution figures. If authors have used the free BioRender service, they can use the instructions provided in the link above to download a high-resolution version suitable for publication.

The link provided should only be used for the purposes of this submission. Authors will be charged for figures created on this account if they are not related to this manuscript submission.

LANGUAGE EDITING AND SUPPORT FOR PUBLICATION: If you would like help with English language editing, or other article preparation support, Wiley Editing Services offers expert help, including English Language Editing, as well as translation, manuscript formatting, and figure formatting at www.wileyauthors.com/eeo/preparation. You can also find resources for Preparing Your Article for general guidance about writing and preparing your manuscript at www.wileyauthors.com/eeo/prepresources.

RIGOUR AND REPRODUCIBILITY: We recommend authors consult The Journal's Rigour and Reproducibility requirements to ensure that their manuscript is suitable for publication: https://physoc.onlinelibrary.wiley.com/pb-assets/hub-assets/physoc/documents/TJP-Rigour-and-Reproducibility-Requirements.pdf

REVISION CHECKLIST:

Check that your Methods section conforms to journal policy: https://jp.msubmit.net/cgi-bin/main.plex?form_type=display_requirements#methods.

Check that data presented conforms to the statistics policy: https://jp.msubmit.net/cgi-bin/main.plex?form_type=display_requirements#statistics.

Upload a full Response to Referees file. To create your 'Response to Referees': copy all the reports, including any comments from the Senior and Reviewing Editors, into a Microsoft Word, or similar, file and respond to each point, using font or background colour to distinguish comments and responses and upload as the required file type.

Please upload two versions of your manuscript text: one with all relevant changes highlighted and one clean version with no changes tracked. The manuscript file should include all tables and figure legends, but each figure/graph should be uploaded as separate, high-resolution files.

You may also upload:

- 'Potential Cover Art' for consideration as the issue's cover image
- Appropriate Supporting Information (Video, audio or data set: see https://jp.msubmit.net/cgi-bin/main.plex?form_type=display_requirements#supp).

We look forward to receiving your revised submission.

If you have any queries, please reply to this email and we will be pleased to advise.

Yours sincerely,

Harold Schultz
Senior Editor
The Journal of Physiology

REQUIRED ITEMS

- Author photo and profile. First or joint first authors are asked to provide a short biography (no more than 100 words for one author or 150 words in total for joint first authors) and a portrait photograph. These should be uploaded and clearly labelled together in a Word document with the revised version of the manuscript. See Information for Authors for further details.

- Please upload separate high-quality figure files via the submission form.

- Papers must comply with the Statistics Policy: https://jp.msubmit.net/cgi-bin/main.plex?form_type=display_requirements#statistics.

In summary:

- If n {less than or equal to} 30, all data points must be plotted in the figure in a way that reveals their range and distribution. A bar graph with data points overlaid, a box and whisker plot or a violin plot (preferably with data points included) are acceptable formats.

- If n > 30, then the entire raw dataset must be made available either as supporting information, or hosted on a not-for-profit repository, e.g. FigShare, with access details provided in the manuscript.

- 'n' clearly defined (e.g. x cells from y slices in z animals) in the Methods. Authors should be mindful of pseudoreplication.

- All relevant 'n' values must be clearly stated in the main text, figures and tables.

- The most appropriate summary statistic (e.g. mean or median and standard deviation) must be used. Standard Error of the Mean (SEM) alone is not permitted.

- Exact p values must be stated. Authors must not use 'greater than' or 'less than'. Exact p values must be stated to three significant figures even when 'no statistical significance' is claimed.

- A Data Availability Statement is required for all papers reporting original data. This must be in the Additional Information section of the manuscript itself. It must have the paragraph heading 'Data Availability Statement'. All data supporting the results in the paper must be either: in the paper itself; uploaded as Supporting Information for Online Publication; or archived in an appropriate public repository. The statement needs to describe the availability or the absence of shared data. Authors must include in their statement: a link to the repository they have used, or a statement that it is available as Supporting Information; reference the data in the appropriate sections(s) of their manuscript; and cite the data they have shared in the References section. Whenever possible, the scripts and other artefacts used to generate the analyses presented in the paper should also be publicly archived. If sharing data compromises ethical standards or legal requirements then authors

are not expected to share it, but must note this in their statement. For more information, see our Statistics Policy.

- Please include an Abstract Figure file, as well as the Figure Legend text within the main article file. The Abstract Figure is a piece of artwork designed to give readers an immediate understanding of the research and should summarise the main conclusions. If possible, the image should be easily 'readable' from left to right or top to bottom. It should show the physiological relevance of the manuscript so readers can assess the importance and content of its findings. Abstract Figures should not merely recapitulate other figures in the manuscript. Please try to keep the diagram as simple as possible and without superfluous information that may distract from the main conclusion(s). Abstract Figures must be provided by authors no later than the revised manuscript stage and should be uploaded as a separate file during online submission labelled as File Type 'Abstract Figure'. Please also ensure that you include the figure legend in the main article file. All Abstract Figures should be created using BioRender. Authors should use The Journal's premium BioRender account to export high-resolution images. Details on how to use and access the premium account are included as part of this email.

- The corresponding author must provide an institutional email address (not a personal address) for their author account. We encourage ALL co-authors to also provide institutional email addresses. If this cannot be provided (as corresponding author), then a stamped letter must be provided from the institution which confirms their role and employment there (please upload this with the revised submission).

EDITOR COMMENTS

Reviewing Editor:

This is a innovative study that has provided some unique findings about the regulation of brain blood flow during running exercise. Although we only secured one expert reviewer, who provided some excellent feedback, i have also provided some additional comments below. Please look to address our comments in a revised manscript.

1. Similar to the reviewer, i find the the findings of a progressive increased in MCAv during running to be very exciting and helps add valuable information to this field. As you note, during these running conditions, CBF may passively follow BP and due to changes in reactivity, may offset the normal (powerful) influence of hypocapnia. It is unfortunate that BP was not measured; however, how much would you anticipant BP to increase by (based on previous studies) during running to exhaustion vs the relative increase in MCAv? it would be useful to highlight this, especially in the context of some buffering of perfusion (autoregulation).

2. Please comment on what you think the fundamental mechanisms of what is causing the blunted cerebrovascular responsiveness to hypocapnia? You mention about CVR has previously been shown to decrease during head-up tilt and increase during ganglionic blockade, which is attributed to sympathetic activation and inhibition (Jordan et al. 2000). However, this study was confounded by PaCO2 changes (unless you mean their 1998 Hypertension 32, 699-704 study?), and many other blockade studies have shown no influence on CVR. For example, data also indicates that the CVR is unaltered (Stroke 7: 507-510, 1976) or augmented by ganglionic blockade or SNA stimulation (J Physiol 551, 609-616.) Please provide a more balance overview on the role of the sympathetic nervous system in cerebrovascular control.

3. Although the methodological limitations is nicely done, what is missing is a clear summary of the end-tidal to arterial differences that will likely be augmented with progress exercise (see Jones NL et al, JAP 1979 for initial study). Such differences will likely be augmented in your study during running in your fit cohort. Please comment on this methodological limitation as is is the PaCO2 that is the 'stimulus' for CVR not end-tidal PCO2.

Please also see 'Required Items' above.

Senior Editor:

Comments for Authors to ensure the paper complies with the Statistics Policy (Required):

Please indicate how summery data is defined throughout the manuscript. The Journal requires summary data as mean(SD) or median with quartiles and confidence intervals. Please define summary data in the data analysis section and in table/figure legends.

The Journal requires actual p values to be provided in tables and figures. When not possible, a supplementary table of the p values made for statistical comparisons in the table/figure needs to be provided. Hence, please provide p values in Figure 6. Please provide supplementary tables of p values for Table 2 and Figure 3.

For uniformity, please indicate sample size and statistical test(s) used in all Tables and Figures in their respective legends. Sample size can be incorporated into a table when possible as desired.

Comments to the Author:
Thank you for submission of your research article to the Journal of Physiology for consideration. The article has been reviewed by experts in the field and found to require a major revision before consideration of acceptability can be determined. Please note that is not a notice of acceptability, which will be dependent upon the adequacy of revision. A major concern raised is uncertainty whether the current protocol adequately addresses the research questions. Referee 1 believes a better protocol would involve comparing CBF oscillation and CVR during heavy exercise, which shows different CBF responses between cycling and running. Therefore, the authors should consider this carefully to validate their conclusions. Please address all comments from the external referee and reviewing editor as well as addressing the list of requirements or publication in the journal including in this letter and the statistical requirements raised herein.

Statistical concerns:

Please indicate how summery data is defined throughout the manuscript. The Journal requires summary data as mean(SD) or median with quartiles and confidence intervals. Please define summary data in the data analysis section and in table/figure legends.

The Journal requires actual p values to be provided in tables and figures. When not possible, a supplementary table of the p values made for statistical comparisons in the table/figure needs to be provided. Hence, please provide p values in Figure 6. Please provide supplementary tables of p values for Table 2 and Figure 3.

For uniformity, please indicate sample size and statistical test(s) used in all Tables and Figures in their respective legends. Sample size can be incorporated into a table when possible as desired.

REFEREE COMMENTS

Referee #1:

This study is intriguing and provides valuable insights into this research area. This study investigated the physiological mechanisms underlying the differing MCA V responses during heavy exercise between these two exercise modes. The authors concluded that this difference is attributed to variations in cerebral CO_2 reactivity rather than differences in cerebral pulsatility. However, I have several serious questions.

Introduction

1) L96-97 "One hypothesis is that these oscillations challenge cerebral autoregulation, leading to the cerebrovasculature becoming more pressure-passive during running."

I find this logic unclear and overly speculative. Cerebral autoregulation serves to reduce cerebral blood flow (CBF) oscillations (damping effect to acute change in BP for maintain adequate CBF operating point). Thus, attenuated cerebral autoregulation would diminish CBF oscillations but should not alter the operating point of CBF. The authors need to provide an explanation, supported by references which provide the evidence that attenuated cerebral autoregulation changes operating point of CBF, for why attenuated cerebral autoregulation would increase the operating point of CBF.

2) Ocillation of CBF

The authors have overlooked important findings regarding the effect of CBF oscillations on endothelin function (J Appl Physiol. 2020 Sep 1;129(3):603-611. doi: 10.1152/japplphysiol.00274.2020). A previous study demonstrated that oscillations increase shear rate and enhance cerebral flow-mediated dilation (FMD). Therefore, running-induced CBF oscillations may elevate cerebral shear rate, which could subsequently increase CBF. This reasoning aligns more closely with the authors' hypothesis, in my view. I think that, at the very least, the authors should not disregard this critical finding in the context of this study.

3) L111-113 "While CO2 reactivity is reported to be maintained or even augmented during cycling exercise, compared to rest (Murrell et al. 2013; Nowak-Flück et al. 2018; Ogoh et al. 2008; Rasmussen et al. 2006), CO2 reactivity during running exercise has never been assessed."

These previous studies did not demonstrate CVR during heavy exercise. The question is about the difference in CBF response during heavy of exhaustive exercise between cycling and running. Thus, the authors should focus on CVR during heavy exercise because CVR during moderate exercise is not related to CBF response to heavy execise. Only Rasmussen et al. may show CVR for hypocapnia and hypercapnia during heavy execise. Thus, the authors should explain this study in more detail to show that no study presented CVR during heavy exercise.

Methods

4) Protocol 2

5) The authors employed 70% VO2max exercise intensity in their study. They should provide a rationale for choosing this intensity. MCA V did not decrease (still increase) at moderate intensity even during cycling, suggesting that this condition may not be suitable for examining the effect of oscillations on MCA V response. The authors should investigate the effects of heavy or exhaustive exercise intensity, with or without CBF oscillations, on the operating point of CBF, as there was no change in CBF response between running and cycling at moderate intensity. This protocol may not fully address the hypothesis. Therefore, the authors should carefully explain how this protocol can effectively address their hypothesis and provide supporting evidence. Additionally, the differences in oscillation observed may not align with the differences in oscillation between heavy running and cycling exercise (i.e., the difference is at a low level). The authors should provide evidence demonstrating that these differences in oscillation are sufficient to account for the distinct effects observed in running and cycling exercises.

6) Protocol 3

I could not find the exercise workload used for measuring CVR, as moderate-intensity exercise increases CBF even during cycling. The authors should compare CVR at high intensity between different exercise modes. I may have missed this information, so please clarify it.

7) Figure 1

The authors hypothesized that cerebral autoregulation interacts with pulsatile CBF to affect the operating point of CBF. If this is the case, I do not understand why the authors introduced the concept of MCA V oscillation. Instead, I believe the authors should focus on BP oscillation and the frequency power of BP that lies outside the range where cerebral autoregulation is effective (approximately up to 0.15 Hz). For the hypothesis to hold, the authors should provide evidence that BP oscillations exceed this range, at the very least.

Discussion

This is an interesting study, but I believe the protocol may not fully address the hypotheses. Therefore, the authors should carefully explain and provide evidence that these protocols are appropriate for testing the hypotheses. I think a better protocol would involve comparing CBF oscillation and CVR during heavy exercise, which shows different CBF responses between cycling and running. I am uncertain whether the current protocol adequately addresses their research questions. If this remains questionable, the results may not provide meaningful insights, and the conclusions could be called into question. Therefore, the authors should explain this carefully to validate their conclusions.

With respect, I believe the research question is very interesting, and this manuscript has the potential to provide valuable information in this research area, provided the validity of the conclusions is ensured.

END OF COMMENTS

In response to the reviewers' comments, we performed two additional experimental protocols to investigate the mechanism underlying the attenuation in cerebrovascular reactivity to hypocapnia we observed during high-intensity running. The findings from our original study demonstrated that MCAv reactivity to hypocapnia was markedly blunted during running. This led to an alternative hypothesis that the apparent loss of cerebrovascular responsiveness during running reflected a limited cerebral vasoconstrictor reserve due to high prevailing arterial pressure and greater static autoregulatory vasoconstriction. To test this hypothesis, we first assessed MCAv responsiveness to hypocapnia at rest and during three progressively higher steady-state cycling workloads (n = 5) to examine the independent effects of exercise intensity and arterial pressure. Second, we compared MCAv responsiveness to hypocapnia during steady-state running and cycling at workloads matched for mean arterial pressure (n = 6) and thus 'autoregulatory load'. Collectively, these additional experiments demonstrated that MCAv responsiveness to hypocapnia decreased with increasing exercise intensity but was similar between running and cycling when mean arterial pressure was matched between modalities. These findings suggest that the attenuation in cerebrovascular responsiveness is related to the prevailing arterial pressure rather than exercise modality *per se and* may reflect level of autoregulatory vasoconstriction.

The manuscript has been revised throughout to incorporate these additional experiments, and our responses to the individual reviewer comments are provided below

EDITOR COMMENTS

Reviewing Editor:

This is a innovative study that has provided some unique findings about the regulation of brain blood flow during running exercise. Although we only secured one expert reviewer, who provided some excellent feedback, i have also provided some additional comments below. Please look to address our comments in a revised manuscript.

1. Similar to the reviewer, i find the findings of a progressive increased in MCAv during running to be very exciting and helps add valuable information to this field. As you note, during these running conditions, CBF may passively follow BP and due to changes in reactivity, may offset the normal (powerful) influence of hypocapnia. It is unfortunate that BP was not measured; however, how much would you anticipant BP to increase by (based on previous studies) during running to exhaustion vs the relative increase in MCAv? it would be useful to highlight this, especially in the context of some buffering of perfusion (autoregulation).

There are limited studies reporting BP increases during incremental running exercise, but it is generally accepted that after very mild intensity exercise, MAP increases linearly with intensity. We have non-invasive BP data from sub-set of participants (n=11) at maximal running exercise during the incremental test. This was not included in the original manuscript as we were unable to reliably collect this data in all participants. In this sub-set, MAP increased by 23±15mmHg from rest to max exercise (Standing Rest MAP: 93±11mmHg Max exercise: 116±9mmHg) while mean MCAv increased by 20cm/sec. Although the near 1:1 ratio between the increase in MAP (i.e., perfusion pressure) and MCAv may suggest passive pressure transmission, we interpret this as indirect evidence that autoregulation remains functional, as other mechanisms contributing to exercise-induced CBF increases (ie., increased neural activity) likely account for part of the observed MCAv increase. To speculate on a time course of events, at the onset of exercise regional metabolic vasodilation, associated with neuronal activation and central command, increases CBF while cerebral autoregulation simultaneously induces vasoconstriction in non-metabolically active regions to offset the initial increase in blood

pressure. As exercise intensity increases, the metabolic demand is likely to increase only modestly, whereas arterial pressure rises progressively. Consequently, increasingly greater autoregulatory vasoconstriction is required within non-metabolically active regions of the brain to offset the rise in arterial pressure. This process will continue until the maximal vasoconstrictor capacity is reached. Beyond this point, the cerebral circulation will be unresponsive to any vasoconstrictor stimuli, not just reductions in CO2. Because the exercise-induced rise in arterial pressure is primarily determined by absolute oxygen uptake, we speculate that exhausting the vasoconstrictor reserve occurs independently of exercise modality. It is simply more likely to occur during running exercise because the absolute VO2 and BP are likely higher during this modality of exercise. While this framework is speculative, our findings are consistent with this interpretation, and it provides a useful conceptual model for understanding the integrative physiological response to exercise. Therefore, it has been included in a perspectives section in the discussion.

2. Please comment on what you think the fundamental mechanisms of what is causing the blunted cerebrovascular responsiveness to hypocapnia? You mention about CVR has previously been shown to decrease during head-up tilt and increase during ganglionic blockade, which is attributed to sympathetic activation and inhibition (Jordan et al. 2000). However, this study was confounded by PaCO2 changes (unless you mean their 1998 Hypertension 32, 699-704 study?), and many other blockade studies have shown no influence on CVR. For example, data also indicates that the CVR is unaltered (Stroke 7: 507-510, 1976) or augmented by ganglionic blockade or SNA stimulation (J Physiol 551, 609-616.) Please provide a more balance overview on the role of the sympathetic nervous system in cerebrovascular control.

We apologise for not including a more balanced view of the potential role of sympathetic nervous system in mediating the observed changes in CVR. We have now changed the discussion and have removed any specific reference to role of the sympathetic nervous system on our findings.

We speculate that the blunted cerebrovascular responsiveness to hypocapnia during running reflects a reduced cerebrovascular vasoconstrictor reserve, rather than an impaired intrinsic sensitivity to CO_2. Running, particularly at high intensities, elicits substantial increases in arterial blood pressure, which would be expected to evoke progressive autoregulatory vasoconstriction. As exercise intensity and arterial pressure increases, autoregulatory vasoconstriction would progressively elevate cerebrovascular tone, taking the cerebral vasculature closer to its vasoconstrictor limit. Under these conditions, the capacity for additional vasoconstriction in response to hypocapnia may be reduced, resulting in an attenuated cerebrovascular response.

We speculate that this effect may be more pronounced during running than cycling because running typically elicits a greater blood pressure response at a given relative intensity. This would place the cerebral vasculature at a higher level of autoregulatory tone, and thus closer to its available vasoconstrictor reserve. Thus, we do not consider this to be a modality-specific phenomenon *per se*, but rather one determined by the prevailing BP. Consistent with this interpretation, we demonstrate that cerebrovascular responsiveness to hypocapnia progressively declines with increasing exercise intensity and blood pressure, with no difference observed when running and cycling are matched for BP. Furthermore, we have unpublished data in two professional cyclists (who have a high modality specific VO2 and thus likely BP) who did not observe a decrease in MCAvmean during an incremental cycling test, suggesting that this lack of reduction in MCAV mean at high intensity exercise, despite reducing end-tidal CO2, may not be modality specific.

We have amended the discussion to provide speculation on the potential mechanism for reduced hypocapnic responsiveness.

3. Although the methodological limitations is nicely done, what is missing is a clear summary of the end-tidal to arterial differences that will likely be augmented with progress exercise (see Jones NL et al, JAP 1979 for initial study). Such differences will likely be augmented in your study during running in your fit cohort. Please comment on this methodological limitation as is is the PaCO2 that is the 'stimulus' for CVR not end-tidal PCO2.

We apologise for not acknowledging this limitation in the original manuscript. The reviewer is correct that compared to rest, the $PETCO_2$-PaCO2 difference may increase during exercise, due to increasing tidal volume (VT) and metabolic CO_2 production (Jones et al., 1979). Potentially, this could have led to an overestimation of the change in $PaCO_2$ based on change in ETCO2 during the reactivity test, resulting in an underestimation of CVR during exercise. As a result, we have estimated $PaCO_2$, in a subset of participants who had both end-tidal gases and respiratory flow data, using validated correction equation provided Jones et al., 1979 and recalculated CVR index. This changes CVR minimally and does not impact our results. CVR remains significantly reduced by from rest to running exercise, regardless of whether ETCO2 or estimated PaCO2 is used in the calculation.

Text added: Third, $ETCO_2$ was used as a surrogate for $PaCO_2$. The end-tidal to arterial PCO_2 difference may widen during exercise with increases in tidal volume and metabolic CO_2 production (Jones et al., 1979, Robbins et al., 1990). This could have led to an overestimation of the change in $PaCO_2$ during exercise and affected our estimation of CVR. To account for this, we estimated arterial PCO_2 using a validated corrective equation (Jones et al., 1979) and calculated CVR. Regardless of whether $ETCO_2$ or estimated $PaCO_2$ was used, cerebrovascular reactivity to hypocapnia was reduced during running exercise (ΔMCAv/$\Delta ETCO_2$; Rest: 1.00±0.27 cm·sec^{-1}/ mmHg, Running: 0.32±0.24 cm·sec^{-1}/ mmHg;

	Hypocapnia trial (n=8)		***P value***
	Rest	Exercise	
**MCAv (cm·sec^{-1})**	Δ-14.1 ± 6.7	Δ-3.3 ± 2.3	**0.0013**
**$ETCO_2$ (mmHg)**	Δ-13.6 ± 5.5	Δ-10.9 ± 5.3	0.14
**Estimated $PaCO_2$ mmHg**	Δ-12.8 ± 5.6	Δ-9.2 ± 4.9	**0.056**
Using $PETCO_2$			
**CVR index (cm·sec^{-1}/ mmHg)**	1.00 ± 0.27	0.32 ± 0.24	**0.003**
**CVR index (%/mmHg)**	2.05 ± 4.67	0.54 ± 0.42	**0.0014**
Using estimated PaCO2			
**CVR index (cm·sec^{-1}/ mmHg)**	1.08 ± 0.30	0.37 ± 0.27	**0.0042**
**CVR index (%/mmHg)**	2.22 ± 0.52	0.62 ± 0.47	**0.002**

ΔMCAv/ΔEstimated$PaCO_2$; Rest: 1.08 ± 0.30 cm·sec^{-1}/ mmHg, Running: 0.37 ± 0.27 cm·sec^{-1}/ mmHg).

Please also see 'Required Items' above.

Senior Editor:

Comments to the Author:
Thank you for submission of your research article to the Journal of Physiology for consideration. The article has been reviewed by experts in the field and found to require a major revision before consideration of acceptability can be determined. Please note that is not a notice of acceptability, which will be dependent upon the adequacy of revision. A major concern raised is uncertainty whether the current protocol adequately addresses the research questions. Referee 1 believes a better protocol would involve comparing CBF oscillation and CVR during heavy exercise, which shows different CBF responses between cycling and running. Therefore, the authors should consider this carefully to validate their conclusions. Please address all comments from the external referee and reviewing editor as well as addressing the list of requirements or publication in the journal including in this letter and the statistical requirements raised herein.

Statistical concerns:

Please indicate how summery data is defined throughout the manuscript. The Journal requires summary data as mean(SD) or median with quartiles and confidence intervals. Please define summary data in the data analysis section and in table/figure legends.

Apologises for not making this clear in the original manuscript. The data are presented as mean values (+-SD). This information has now been added to the manuscript.

The Journal requires actual p values to be provided in tables and figures. When not possible, a supplementary table of the p values made for statistical comparisons in the table/figure needs to be provided. Hence, please provide p values in Figure 6. Please provide supplementary tables of p values for Table 2 and Figure 3.

P values have now been added to Figure 6 and supplementary tables have been added to now include p values for Table 2 and Figure 3.

For uniformity, please indicate sample size and statistical test(s) used in all Tables and Figures in their respective legends. Sample size can be incorporated into a table, when possible, as desired.

This information has now been added to the manuscript.

REFEREE COMMENTS

Referee #1:

This study is intriguing and provides valuable insights into this research area. This study investigated the physiological mechanisms underlying the differing MCA V responses during heavy exercise between these two exercise modes. The authors concluded that this difference is attributed to variations in cerebral CO2 reactivity rather than differences in cerebral pulsatility. However, I have several serious questions.

We would like to clarify that the paper wasn't initially supposed to be a direct comparison between cycling and running. It was originally designed to understand why MCAv mean progressively increased during higher intensity running exercise and did not show the typical reduction in MCA vmean dispute reducing ETCO2. We appreciate that the wording we've used throughout may have made it appear this way. Nonetheless based on the reviewer comments we supplemented this data with additional experiments to directly compare running versus cycling, importantly at fixed blood pressures and not relative exercise intensities.

Introduction

1) L96-97 "One hypothesis is that these oscillations challenge cerebral autoregulation, leading to the cerebrovasculature becoming more pressure-passive during running."

I find this logic unclear and overly speculative. Cerebral autoregulation serves to reduce cerebral blood flow (CBF) oscillations (damping effect to acute change in BP for maintain adequate CBF operating point). Thus, attenuated cerebral autoregulation would diminish CBF oscillations but should not alter the operating point of CBF. The authors need to provide an explanation, supported by references which provide the evidence that attenuated cerebral autoregulation changes operating point of CBF, for why attenuated cerebral autoregulation would increase the operating point of CBF.

We can agree the logic is simplistic, however, this was a hypothesis proposed by several studies (Furlong et al. 2020; Lyngeraa et al. 2013). The rationale was that the cyclic BP oscillations during running impose a continuous dynamic autoregulatory demand, which may limit the capacity of the cerebrovasculature to simultaneously buffer the slower, intensity-dependent rise in mean arterial pressure. Under such conditions, progressive increases in perfusion pressure would theoretically be transmitted more directly to the cerebral circulation, rather than being effectively offset by static autoregulatory vasoconstriction. We acknowledge that this mechanism is highly speculative and that is why we tested it by manipulating these pulsatility oscillations during high intensity running.

Importantly, despite attenuating the oscillatory pulsatility profile, MCAvmean was unchanged. Thus, our data do not support the hypothesis.

2) Ocillation of CBF

The authors have overlooked important findings regarding the effect of CBF oscillations on endothelin function (J Appl Physiol. 2020 Sep 1;129(3):603-611. doi: 10.1152/japplphysiol.00274.2020). A previous study demonstrated that oscillations increase shear rate and enhance cerebral flow-mediated dilation (FMD). Therefore, running-induced

CBF oscillations may elevate cerebral shear rate, which could subsequently increase CBF. This reasoning aligns more closely with the authors' hypothesis, in my view. I think that, at the very least, the authors should not disregard this critical finding in the context of this study.

We thank the reviewer for raising this important point and agree that oscillatory flow patterns may influence cerebrovascular function through shear-mediated mechanisms. The study cited provides evidence that oscillations in cerebral blood flow/mean velocity can increase shear and enhance flow-mediated dilation. However, we believe there is an important physiological distinction between the oscillatory flow profile induced during intermittent hypoxia and the pulsatility oscillations observed during running.

In the intermittent hypoxia model, oscillations occur in mean velocity, where both systolic and diastolic velocities must be increasing/decreasing together, resulting in cyclic changes in mean shear, as mean velocity is a primary determinant of shear. In contrast, during running the oscillations are in pulsatility, not mean velocity, whereby increases in systolic velocity are accompanied by simultaneous decreases in diastolic velocity (and vice versa), such that mean velocity remains relatively stable over time. As mean velocity is the primary determinant of shear rate, this suggests that the net shear stimulus may not be substantially altered despite large beat-to-beat fluctuations in pulsatility.

This interpretation is further supported by our foot-strike manipulation data. Whilst altering foot-strike timing significantly changed the pulsatility profile, MCAv mean remained unchanged across the free-running (pulsatility oscillations), diastolic stepping (reduced oscillations but high pulsatility), systolic stepping (reduced oscillations but low pulsatility) conditions. If pulsatility-driven shear was a major determinant of the elevated MCAv response during running, reducing these oscillations would be expected to alter MCAv mean, which was not observed. Therefore, whilst we acknowledge that shear-mediated mechanisms may contribute to cerebrovascular regulation, our data suggest that the oscillatory pulsatility profile observed during running is unlikely to directly increase cerebral blood flow via enhanced shear-mediated dilation. We have now acknowledged and discussed this distinction in the revised manuscript.

We discuss the potential role of shear-mediated mechanism in the methodological considerations and we have now added a sentence acknowledging this in the introduction

Text added to introduction: These oscillations in pulsatility may also have downstream effects on shear-mediated mechanisms.

3) L111-113 "While CO2 reactivity is reported to be maintained or even augmented during cycling exercise, compared to rest (Murrell et al. 2013; Nowak-Flück et al. 2018; Ogoh et al. 2008; Rasmussen et al. 2006), CO2 reactivity during running exercise has never been assessed."

These previous studies did not demonstrate CVR during heavy exercise. The question is about the difference in CBF response during heavy of exhaustive exercise between cycling and running. Thus, the authors should focus on CVR during heavy exercise because CVR during moderate exercise is not related to CBF response to heavy execise. Only Rasmussen et al. may show CVR for hypocapnia and hypercapnia during heavy execise. Thus, the authors should explain this study in more detail to show that no study presented CVR during heavy exercise.

Most previous studies assessing cerebrovascular CO_2 reactivity during cycling have been performed at low to moderate exercise intensities, and importantly in our opinion relatively low

fit individuals. Thus we agree that these findings cannot be extrapolated to heavy or exhaustive exercise. Importantly, there was previously no literature examining how cerebrovascular CO_2 reactivity changes across increasing exercise intensities but, in a follow-up study, we demonstrate that cerebrovascular responsiveness to hypocapnia appears to progressively decrease with increasing exercise intensity, confirming that cerebrovascular CO_2 responsiveness to hypocapnia during moderate exercise cannot be assumed to reflect responses during heavy exercise. Rasmussen et al. is the only study to our knowledge that assessed hypocapnic reactivity during higher-intensity cycling exercise (~67% $\dot{V}O_2$max), but this was integrated into an overall reactivity slope alongside hypercapnia. We have revised the introduction to clarify this distinction and avoid overstating the current literature. We now emphasise that cerebrovascular CO_2 reactivity during heavy exercise, particularly during running, remains poorly characterised. Moreover, we have highlighted that our first protocols were always in an intensity domain where CO2 is reduced (i.e. above the 2nd ventiltory threshold).

Text added: In contrast, only two studies have examined MCAv reactivity to hypocapnia during exercise (Rasmussen et al. 2006; Ogoh et al., 2008). Ogoh et al., (2008) reported no difference in hypocapnic reactivity during low intensity cycling (32±2% VO2 max). However, these findings are likely not representative of cerebrovascular regulation at higher intensities where increases in BP and hypocapnia become more pronounced. Rasmussen et al. (2006), who studied participants during moderate intensity cycling (~67% $\dot{V}O_2$max), reported an increased cerebrovascular CO2 reactivity. However, Rasmussen and colleagues (2006) derived a single MCAv CO2 reactivity curve MCAv by integrating the hypercapnic and hypocapnic responses using a double-logarithmic regression analysis. While the combined analysis suggested an improved cerebrovascular CO2 reactivity, visual inspection of their data indicates increased hypercapnic reactivity but decreased hypocapnic reactivity.

Methods

4) Protocol 2

5) The authors employed 70% VO2max exercise intensity in their study. They should provide a rationale for choosing this intensity. MCA V did not decrease (still increase) at moderate intensity even during cycling, suggesting that this condition may not be suitable for examining the effect of oscillations on MCA V response. The authors should investigate the effects of heavy or exhaustive exercise intensity, with or without CBF oscillations, on the operating point of CBF, as there was no change in CBF response between running and cycling at moderate intensity. This protocol may not fully address the hypothesis. Therefore, the authors should carefully explain how this protocol can effectively address their hypothesis and provide supporting evidence. Additionally, the differences in oscillation observed may not align with the differences in oscillation between heavy running and cycling exercise (i.e., the difference is at a low level). The authors should provide evidence demonstrating that these differences in oscillation are sufficient to account for the distinct effects observed in running and cycling exercises.

The reviewer raises an important point, and we apologise that this was not made explicitly clear/justified in the original manuscript. During pilot testing, it was practically difficult for individuals to match their foot-strike to a metronome at near maximal exercise, so we decided to use a lower intensity, but importantly one that was above the exercise intensity where the responses between cycling and running are diverging (~60% VO2max). This workload corresponded to 70.9±9.0% VO_2max, which was chosen as this is an intensity where $ETCO_2$

is falling (i.e. above the 2nd ventilatory threshold), thus MCAv would be expected to be decreasing. Indeed, it is important to recognize that running above the 2nd ventilatory threshold in the produces modest hypocapnia compared to the same relative intensity during the VO2max test. i.e. ETCO2 is reduced roughly 32 mmHg, which is close to values observed at VO2max. Furthermore, pulsatility oscillations occurred at 0.22Hz during this workload, which is above the range where dynamic autoregulation is considered effective. Therefore, we believe that the exercise intensity is appropriate to interrogate the question as to why MCAv continues to increase, despite reductions in ETCO2.

Text added: During free running, the pulsatility oscillation frequency was 0.22±0.04Hz with all participants exhibiting oscillation frequencies above 0.15Hz, which is the upper range where dynamic cerebral autoregulation is most effective.

6) Protocol 3

I could not find the exercise workload used for measuring CVR, as moderate-intensity exercise increases CBF even during cycling. The authors should compare CVR at high intensity between different exercise modes. I may have missed this information, so please clarify it.

Thank you for the comment and apologises for not presenting this data in the original manuscript. The exercise intensity was 76±4% VO2 max, but individualised based on individual RCP on incremental test during visit 1. Importantly, this workload was when ETCO2 was decreasing (~31 mmHg), thus MCAv would be expected to be decreasing.

7) Figure 1

The authors hypothesized that cerebral autoregulation interacts with pulsatile CBF to affect the operating point of CBF. If this is the case, I do not understand why the authors introduced the concept of MCA V oscillation. Instead, I believe the authors should focus on BP oscillation and the frequency power of BP that lies outside the range where cerebral autoregulation is effective (approximately up to 0.15 Hz). For the hypothesis to hold, the authors should provide evidence that BP oscillations exceed this range, at the very least.

We apologise for the confusion. The MCAv oscillation index was included solely as a quantitative index of cerebrovascular pulsatility and to confirm that altering foot-strike timing was an effective intervention for reducing the oscillatory pattern. It was not intended to provide information regarding the frequency of BP oscillations or the operating range of cerebral autoregulation.

The hypothesis that foot-strike-related oscillations may impair autoregulatory buffering was based on previous studies that reported these oscillations occur at frequencies above the range over which cerebral autoregulation is most effective (~0.15 Hz). In protocol 2, we assessed the effect of removing oscillations, the BP oscillation frequency during the free-running condition, was 0.22 Hz, exceeding this range.

Unfortunately, beat-to-beat BP was not measured during the incremental running test, and therefore we cannot quantify BP oscillation frequency across exercise intensities, but previous studies have reported that running induced BP oscillations do consistently exceed 0.15Hz at several workloads up to 75% HR reserve.

Text added: MCA pulsatility variability was used to numerically quantify the beat-by-beat oscillations in pulsatility during running (Figure 1) to confirm whether foot-strike manipulation successfully reduced pulsatility oscillations.

Discussion

This is an interesting study, but I believe the protocol may not fully address the hypotheses. Therefore, the authors should carefully explain and provide evidence that these protocols are appropriate for testing the hypotheses. I think a better protocol would involve comparing CBF oscillation and CVR during heavy exercise, which shows different CBF responses between cycling and running. I am uncertain whether the current protocol adequately addresses their research questions. If this remains questionable, the results may not provide meaningful insights, and the conclusions could be called into question. Therefore, the authors should explain this carefully to validate their conclusions.

With respect, I believe the research question is very interesting, and this manuscript has the potential to provide valuable information in this research area, provided the validity of the conclusions is ensured.

We thank the reviewer for this important point and perhaps the disagreement is related to how we have described the exercise intensities were chosen, because in agreement with the reviewer, our protocols were specifically designed to assess cerebrovascular control at workloads in the intense domain where $ETCO_2$ had already declined to near maximal values, as this is the point at which MCAv would normally be expected to decrease if cerebrovascular CO_2 reactivity remained intact. This is also above the exercise domain where the divergence in CBF responses between running and cycling has previously been reported.

Accordingly, the cerebrovascular CO_2 reactivity protocol was performed at a workload above the respiratory compensation point, where MCAv would be expected to be falling. Instead, MCAv was maintained, allowing us to directly interrogate the mechanisms underlying this dissociation.

At this workload, hypocapnic responsiveness was already markedly blunted, with the reduction in MCAv during hypocapnia almost abolished. While it could be argued that assessing CVR at a higher exercise intensity may have strengthened this observation, our additional data demonstrate that cerebrovascular responsiveness to hypocapnia progressively decreases with increasing exercise intensity. Therefore, a higher workload would be expected to further exaggerate this blunting rather than alter our interpretation.

Similarly, we believe the foot-strike manipulation protocol was appropriate for testing the pulsatility hypothesis. The selected workload produced a dominant oscillation frequency of 0.22 Hz, which lies above the frequency range over which cerebral autoregulation is considered most effective (~0.15 Hz). Thus, the physiological conditions were sufficient to test whether foot-strike-related oscillations could impair autoregulatory buffering and alter MCAvmean. We acknowledge that oscillation frequency may vary with exercise intensity, likely due to the interaction of heart rate and stride frequency. However, based on MCAv pulsatility during the incremental exercise test, the greatest oscillatory amplitudes - where any impairment of autoregulatory buffering might be expected to be greatest - were often observed at lower exercise intensities, where no divergence in MCAvmean between running and cycling has been reported. This is consistent with our findings that these oscillations do not appear to

influence mean MCAv. Importantly, despite experimentally reducing foot-strike-related oscillations at a workload where the dominant oscillation frequency was already outside the effective autoregulatory range, MCAvmean remained unchanged. Together, these findings suggest that foot-strike-related pulsatility is unlikely to meaningfully alter the operating point of CBF under these conditions.

Dear Dr Simpson,

Re: JP-RP-2026-288117R1 "Mechanisms regulating cerebral perfusion during running: Influence of foot-strike induced pulsatility oscillations, CO2 reactivity and blood pressure" by Lydia L Simpson, Dean R Perkins, Connor A Howe, Christina Dominique Bruce, William K Cornwell III, and Justin Stevan Lawley

Thank you for submitting your manuscript to The Journal of Physiology. It has been assessed by a Reviewing Editor and by 1 expert referee and we are pleased to tell you that it is acceptable for publication following satisfactory revision.

Please share this decision with your co-authors as soon as possible.

The referee reports are copied at the end of this email.

Please address all the points raised and incorporate all requested revisions or explain in your Response to Referees why a change has not been made. We hope you will find the comments helpful and that you will be able to return your revised manuscript within 4 weeks. If you require longer than this, please contact journal staff: jp@physoc.org.

Your revised manuscript should be submitted online using the link in your Author Tasks Link Not Available. This link is accessible via your account as Corresponding Author; it is not available to your co-authors. If this presents a problem, please contact journal staff (jp@physoc.org). Image files from the previous version are retained on the system. Please ensure you replace or remove any files that are being revised.

If you do not wish to submit a revised version of your manuscript, you must inform our journal staff (jp@physoc.org) or reply to this email to request withdrawal. Please note that a manuscript must be formally withdrawn from the peer review process at one journal before it may be submitted to another journal.

TRANSPARENT PEER REVIEW POLICY: To improve the transparency of its peer review process The Journal of Physiology publishes online, as supporting information, the peer review history of all articles accepted for publication. Readers will have access to decision letters, including Editors' comments and referee reports, for each version of the manuscript, as well as any author responses to peer review comments. Referees can decide whether or not they wish to be named on the peer review history document.

ABSTRACT FIGURES: Authors are expected to use The Journal's premium BioRender account to create/redraw their Abstract Figures. Information on how to access this account is here: https://physoc.onlinelibrary.wiley.com/journal/14697793/biorender-access.

This will enable Authors to create and download high-resolution figures. If authors have used the free BioRender service, they can use the instructions provided in the link above to download a high-resolution version suitable for publication.

The link provided should only be used for the purposes of this submission. Authors will be charged for figures created on this account if they are not related to this manuscript submission.

LANGUAGE EDITING AND SUPPORT FOR PUBLICATION: If you would like help with English language editing, or other article preparation support, Wiley Editing Services offers expert help, including English Language Editing, as well as translation, manuscript formatting, and figure formatting at www.wileyauthors.com/eeo/preparation. You can also find resources for Preparing Your Article for general guidance about writing and preparing your manuscript at www.wileyauthors.com/eeo/prepresources.

RIGOUR AND REPRODUCIBILITY: We recommend authors consult The Journal's Rigour and Reproducibility requirements to ensure that their manuscript is suitable for publication: https://physoc.onlinelibrary.wiley.com/pb-assets/hub-assets/physoc/documents/TJP-Rigour-and-Reproducibility-Requirements.pdf

REVISION CHECKLIST:

Check that your Methods section conforms to journal policy: https://jp.msubmit.net/cgi-bin/main.plex?form_type=display_requirements#methods

Check that data presented conforms to the statistics policy: https://jp.msubmit.net/cgi-bin/main.plex?form_type=display_requirements#statistics

Upload a full Response to Referees file. To create your 'Response to Referees' copy all the reports, including any comments from the Senior and Reviewing Editors, into a Microsoft Word, or similar, file and respond to each point, using font or background colour to distinguish comments and responses and upload as the required file type.

Please upload two versions of your manuscript text: one with all relevant changes highlighted and one clean version with no changes tracked. The manuscript file should include all tables and figure legends, but each figure/graph should be uploaded

as separate, high-resolution files. The journal is now integrated with Wiley's Image Checking service. For further details, see: https://www.wiley.com/en-us/network/publishing/research-publishing/trending-stories/upholding-image-integrity-wileys-image-screening-service

You may also upload:

- 'Potential Cover Art' for consideration as the issue's cover image
- Appropriate Supporting Information (Video, audio or data set: see https://jp.msubmit.net/cgi-bin/main.plex?form_type=display_requirements#supp).

We look forward to receiving your revised submission.

If you have any queries, please reply to this email and we will be pleased to advise.

Yours sincerely,

Laura Bennet
Senior Editor
The Journal of Physiology

REQUIRED ITEMS

(1) Your paper contains Supporting Information of a type that we no longer publish, including supplementary tables and figures. Any information essential to an understanding of the paper must be included as part of the main manuscript and figures. The only Supporting Information that we publish are video and audio, 3D structures, program codes and large data files. Your revised paper will be returned to you if it does not adhere to our Supporting Information Guidelines.

(2) Authors are required to fully disclose the use of any Generative AI (GAI) tools used in the preparation of their manuscript. This disclosure should also be included as a statement alongside the Additional Information and Data Availability statements towards the end of the article under the heading 'Generative AI Statement', and include the name(s), model and version of AI technology used, and exact details on the purpose for, and methods of, its use.

If no AI tool has been used, this should also be specifically stated within the manuscript.

EDITOR COMMENTS

Reviewing Editor:

Thank you for your thorough revisions and updates to your manuscript - it adds a important contribution to the understanding of the regulation of brain blood flow during exercise.

Please see 'Required Items' above.

Senior Editor:

Thank you for your resubmission. Please check the following:

The Journal does not permit supplemental information. Please either incorporate the supplemental table 1 into the text or remove all together.

Please provide a statement about the use of AI as per Journal requirements.

3 references have some issues and we request you check that all cited references are correct and cited in the Journal style, which they are not currently.

These references need to be checked for DOI

1. The DOI for this one is incorrect. Edwards, M. R., D. H. Martin, and R. L. Hughson. 2002. "Cerebral Haemodynamics and Resistance Exercise." Medicine & Science in Sports & Exercise 34(7):1207-11. doi: 10.1519/00126548-200202000-00009.

2. The DOI for this paper is incorrect and comes back to a 1993 paper by Dean on the The San Francisco earthquake of 1906. The paper also has the journal information in twice. Giller, CA, G. Bowman, H. Dyer, L. Mootz, and W. Krippner. 1993. "Cerebral Arterial Diameters during Changes in Blood Pressure and Carbon Dioxide during Craniotomy. Neurosurgery 32: 737-741, 1993." Neurosurgery 32:737-41. doi:834

10.1080/00033799300200371

3. This paper has an incorrect DOI and is formatted incorrectly. Zhang, Rong, Julie H. Zuckerman, C. A. Giller, and Benjamin D. Levine. 1998. "Transfer Function Analysis of Dynamic Cerebral Autoregulation in Humans." Am J Physiol Heart Circ Physiol 274(43):233-41. doi: 10.1016/j.preghy.2015.09.001. The DOI comes back to a paper by Keith et al

----

REFEREE COMMENTS

Referee #1:

Thank you for this response. The authors addressed my concerns. I believe that this manuscript provides some important information in this research area.

END OF COMMENTS

REQUIRED ITEMS

(1) Your paper contains Supporting Information of a type that we no longer publish, including supplementary tables and figures. Any information essential to an understanding of the paper must be included as part of the main manuscript and figures. The only Supporting Information that we publish are video and audio, 3D structures, program codes and large data files. Your revised paper will be returned to you if it does not adhere to our Supporting Information Guidelines.

REPLY: This supplementary table has now been removed

(2) Authors are required to [<a]https://jp.msubmit.net/cgi-bin/main.plex?form_type=display_requirements#aigcfully disclose the use of any Generative AI (GAI) tools used in the preparation of their manuscript. This disclosure should also be included as a statement alongside the Additional Information and Data Availability statements towards the end of the article under the heading 'Generative AI Statement', and include the name(s), model and version of AI technology used, and exact details on the purpose for, and methods of, its use.

If no AI tool has been used, this should also be specifically stated within the manuscript.

REPLY: This statement has now been included in the manuscript

EDITOR COMMENTS

Reviewing Editor:

Thank you for your thorough revisions and updates to your manuscript - it adds a important contribution to the understanding of the regulation of brain blood flow during exercise.

Please see 'Required Items' above.

Senior Editor:

Thank you for your resubmission. Please check the following:

The Journal does not permit supplemental information. Please either incorporate the supplemental table 1 into the text or remove all together.

REPLY: This supplementary table has now been removed

Please provide a statement about the use of AI as per Journal requirements.

REPLY: This has now been included in the manuscript

3 references have some issues and we request you check that all cited references are correct and cited in the Journal style, which they are not currently.

REPLY: The references have now been reformatted using Journal of Physiology citation style in EndNote.

These references need to be checked for DOI

1. The DOI for this one is incorrect. Edwards, M. R., D. H. Martin, and R. L. Hughson. 2002. "Cerebral Haemodynamics and Resistance Exercise." Medicine & Science in Sports & Exercise 34(7):1207-11. doi: 10.1519/00126548-200202000-00009.

2. The DOI for this paper is incorrect and comes back to a 1993 paper by Dean on the The San Francisco earthquake of 1906. The paper also has the journal information in twice. Giller, CA, G. Bowman, H. Dyer, L. Mootz, and W. Krippner. 1993. "Cerebral Arterial Diameters during Changes in Blood Pressure and Carbon Dioxide during Craniotomy. Neurosurgery 32: 737-741, 1993." Neurosurgery 32:737-41. doi:834

10.1080/00033799300200371

3. This paper has an incorrect DOI and is formatted incorrectly. Zhang, Rong, Julie H. Zuckerman, C. A. Giller, and Benjamin D. Levine. 1998. "Transfer Function Analysis of Dynamic Cerebral Autoregulation in Humans." Am J Physiol Heart Circ Physiol 274(43):233-41. doi: 10.1016/j.preghy.2015.09.001. The DOI comes back to a paper by Keith et al

REPLY: The references have now been reformatted using Journal of Physiology citation style in EndNote.

Dear Dr Simpson,

Re: JP-RP-2026-288117R2 "Mechanisms regulating cerebral perfusion during running: Influence of foot-strike induced pulsatility oscillations, CO2 reactivity and blood pressure" by Lydia L Simpson, Dean R Perkins, Connor A Howe, Christina Dominique Bruce, William K Cornwell III, and Justin Stevan Lawley

We are pleased to tell you that your paper has been accepted for publication in The Journal of Physiology.

The last Word (or similar) version of the manuscript provided will be used by the Production Editor to prepare your proof. When this is ready you will receive an email containing a link to Wiley's Online Proofing System. The proof should be thoroughly checked and corrected as promptly as possible.

Authors should note that it is too late at this point to offer corrections prior to proofreading. Major corrections at proof stage, such as changes to figures, will be referred to the Editors for approval before they can be incorporated. Only minor changes, such as to style and consistency, should be made at proof stage. Changes that need to be made after proof stage will usually require a formal correction notice.

All queries at proof stage should be sent to: TJP@wiley.com.

Following copyediting and typesetting the accepted version will be published online.

Yours sincerely,

Laura Bennet
Senior Editor
The Journal of Physiology

IMPORTANT POINTS TO NOTE FOLLOWING ACCEPTANCE OF YOUR PAPER:

• IMPORTANT NOTICE ABOUT OPEN ACCESS: To assist authors whose funding agencies mandate immediate public access to published research findings, The Journal of Physiology allows authors to pay an Open Access (OA) fee to have their papers made freely available immediately on publication.

The Corresponding Author will receive an email from Wiley with details on how to register or log in to Wiley Authors where you will be able to place an order.

• You can check if your funder or institution has a Wiley Open Access Account here:
https://authors.wiley.com/author-resources/Journal-Authors/open-access/author-compliance-tool.html

MEMBER DISCOUNT: Corresponding authors who are Members of the Physiological Society are eligible for a 20% discount on the cost of the Article Processing Charge (APC). To claim this discount, the Corresponding Author will need to log in to the Physiological Society Membership portal and retrieve the discount code (https://www.physoc.org/journals-and-media/journals/journal-discount-for-members/). This code should then be quoted in the billing section of Wiley Authors when selecting the open access option.

• TRANSPARENT PEER REVIEW POLICY: To improve the transparency of its peer review process, The Journal of Physiology publishes online as supporting information the peer review history of all articles accepted for publication. Readers will have access to decision letters, including Editors' comments and referee reports, for each version of the manuscript as well as any author responses to peer review comments. Referees can decide whether or not they wish to be named on the peer review history document.

• You can help your research get the attention it deserves! Check out Wiley's free Promotion Guide for best-practice recommendations for promoting your work at: www.wileyauthors.com/eeo/guide. You can learn more about Wiley Editing Services which offers professional video, design, and writing services to create shareable video abstracts, infographics, conference posters, lay summaries, and research news stories for your research at: www.wileyauthors.com/eeo/promotion.

• If you would like to receive our 'Research Roundup', a monthly newsletter highlighting the cutting-edge research published in The Physiological Society's family of journals (The Journal of Physiology, Experimental Physiology, Physiological Reports, The Journal of Nutritional Physiology and The Journal of Precision Medicine: Health and Disease), please click this link, fill in your name and email address and select 'Research Roundup':
https://www.physoc.org/journals-and-media/membernews
